# Barriers and facilitators to collaborative care implementation within the New York State Collaborative Care Medicaid Program

**DOI:** 10.1186/s12913-024-10909-0

**Published:** 2024-04-24

**Authors:** Erin LePoire, Molly Joseph, Ashley Heald, Danielle Gadbois, Amy Jones, Joan Russo, Deborah J Bowen

**Affiliations:** 1 University of Washington AIMS Center, Seattle, WA USA; 2 New York State Office of Mental Health, Albany, NY USA; 3 Department of Psychiatry and Behavioral Sciences, University of Washington, Seattle, WA USA; 4 Department of Bioethics and Humanities, University of Washington, Seattle, WA USA

**Keywords:** Collaborative care, Implementation, Barriers to implementation

## Abstract

**Background:**

Since 2015, the New York State Office of Mental Health has provided state primary care clinics with outreach, free training and technical assistance, and the opportunity to bill Medicaid for the Collaborative Care Model (CoCM) as part of its Collaborative Care Medicaid Program. This study aims to describe the characteristics of New York State primary care clinics at each step of CoCM implementation, and the barriers and facilitators to CoCM implementation for the New York State Collaborative Care Medicaid Program.

**Methods:**

In this mixed-methods study, clinics were categorized into RE-AIM (Reach, Effectiveness, Adoption, Implementation, and Maintenance) steps. Clinics were sent a survey, which included questions related to payer mix, funding sources, billing codes used, and patient population demographics. Qualitative interviews were conducted with clinic representatives, focusing on barriers or facilitators clinics experienced affecting their progression to the next RE-AIM step.

**Results:**

One thousand ninety-nine surveys were sent to primary care clinics across New York State, with 107 (9.7%) completing a survey. Significant differences were observed among the different RE-AIM steps for multiple demographic variables including primary payer, percentage of patients with a diagnose of depression or anxiety, and percent of behavioral health services that are reimbursed, in addition to others. Three main themes regarding barriers and facilitators to implementing CoCM for New York State Medicaid billing emerged from 31 qualitative interviews: (1) Billing requirements, (2) Reimbursement rates, and (3) Buy-in to CoCM.

**Conclusions:**

Survey data align with what we would expect to see demographically in NYS primary care clinics. Qualitative data indicated that CoCM billing requirements/structure and reimbursement rates were perceived as barriers to providing CoCM, particularly with New York State Medicaid, and that buy-in, which included active involvement from organizational leaders and providers that understand the Collaborative Care model were facilitators. Having dedicated staff to manage billing and data reporting is one way clinics minimize barriers, however, there appeared to be a disconnect between what clinics can bill for and the reimbursed amount several clinics are receiving, illustrating the need for stronger billing workflows and continued refinement of billing options across different payers.

**Supplementary Information:**

The online version contains supplementary material available at 10.1186/s12913-024-10909-0.

## Background

The passage of the Affordable Care Act greatly expanded behavioral healthcare coverage and access to many Americans [1], yet the demand for these services far outweighs what can be provided by the existing behavioral health workforce [2]. As such, leaders must look for more creative ways to leverage the skills of specialists and increase access to care. One approach to achieve this is to integrate behavioral healthcare into primary care settings, where individuals typically receive a majority of their healthcare services [3]. Collaborative Care is a model of integrated care that treats common behavioral health conditions such as depression and anxiety, in primary care settings using a team-based approach. Over 90 studies demonstrate that Collaborative Care (CoCM) is more effective than traditional care in treating these disorders [4, 5]. Collaborative Care has been a reimbursable Medicaid service in New York State (NYS) since 2015. Clinics interested in billing Medicaid for CoCM must apply to the NYS Collaborative Care Medicaid Program (CCMP) through the New York State Office of Mental Health (OMH). OMH approves applications and provides free training and technical assistance to clinics interested in implementing CoCM.

Across all payers, Collaborative Care reimbursement is structured as a monthly payment. Medicare reimbursement is based on minutes spent by the behavioral health care manager (BHCM) doing CoCM tasks; whereas NYS Medicaid billing requirements include a documented clinical contact and symptom monitoring tool, along with a visit with a licensed provider in the past 90 days. NYS Medicaid also offers a quality supplemental retainage payment to hospital-affiliated clinics and Federally Qualified Health Centers (FQHCs) to earn an additional 25% reimbursement. Patients are retainage-eligible once they have received three months of Collaborative Care services and either have a) a demonstrable clinical improvement or b) a documented review of the case by the psychiatrist or a documented change to the treatment plan [6].

Medicare billing also varies by practice type. For private practices and hospital-affiliated clinics, there are a variety of different codes and reimbursement rates based on the minutes spent in the initial and subsequent months of treatment. However, FQHCs are much more limited in the codes for which they can bill [7].

As the aforementioned descriptions illustrate, CoCM billing requirements and payment structure for both NYS Medicaid and Medicare are more complex than traditional fee-for-service (FFS) billing, and there are different permissions and restrictions associated with billing among the major primary care clinic types: private practices, hospital-affiliated clinics, and FQHCs. To add to this complexity, the various clinic types have their own rules about what kind of behavioral healthcare can be provided, to whom, and by whom. See Appendix 2 for a breakdown of some of these rules, as well as a comparison of NYS Medicaid, Medicare, and psychotherapy billing based on clinic type. We were interested in these factors and how they may relate to participation in the New York State Collaborative Care Medicaid Program (CCMP).

Previous research has used the Reach, Effectiveness, Adoption, Implementation, and Maintenance (RE-AIM) [8, 9] model to define the steps leading to full participation in the CCMP and to present the proportion of NYS primary care clinics occupying each of these steps as of the year 2019. RE-AIM is a decades-old model used for planning and evaluating the translation of research-based practices into real-world settings [10].

The goal of this paper was to build on previous research and learn more about the specific facilitators and barriers NYS primary care clinics faced when implementing Collaborative Care for Medicaid billing. We aimed to 1) investigate the impact that clinic characteristics (i.e., clinic type, clinic demographics, patient demographics, payer mix, and reimbursement for behavioral health (BH) care might have on implementation and 2) understand the barriers and facilitators contributing to implementation of CoCM through in-depth interviews with clinics at each step of the RE-AIM framework.

## Methods

### Measuring RE-AIM dimensions

The RE-AIM categories were previously established [10] and defined as follows: *Reached* - clinics that had at least one contact with OMH expressing an interest in implementing CoCM but did not go on to receive training and technical assistance (TTA). *Effectiveness* was also defined and reported in the previous paper but is not assessed in this study due to a lack of behavioral health symptom improvement data from clinics that did not implement or maintain CoCM. *Adopted* - clinics that received some form of TTA but did not go on to submit at least one quarter of data to OMH. *Implemented* - clinics that submitted at least one quarter of data but less than four quarters (one year) of data. *Maintained* - clinics that submitted at least one quarter of data for a year or more since their first quarterly report submission. Clinics that never had an initial contact with OMH are categorized as Never Reached.

### Quantitative survey development and administration

A 70-item survey was created specifically for this study (Appendix 6) using REDCap [11] in order to collect a variety of clinical and demographic information about clinics, including 1.) Clinic demographics, 2.) Patient demographics, 3.) Insurance and funding, 4.) and CoCM billing utilization, which is summarized in Table 1. Two datasets were used to create the sampling frame. The first was OMH's internal dataset of clinics with which they have had contact. This served as our sample for clinics falling into the Reached, Adopted, Implemented, and Maintained categories. The second dataset was the New York State Provider Network Data System [12], a publicly available dataset that we used to create our sample of Never Reached clinics. Eligibility criteria for clinics included an address within the boundaries of New York State, and the delivery of primary care services listed as either the first or second type of service provided at the clinic. Clinics were excluded from our contact list if their services were provided primarily via house calls, in a homeless shelter, school, nursing home, or psychiatric clinic. Dental clinics were excluded, as well as clinics that were temporarily or permanently closed during the data collection time period, years 2020 and 2021.

**Table 1:** Survey data organized by RE-AIM step

	TOTAL *N*=107	Never Reached (*N*=17,16%)	Reached (Never Adopted) (*N*=9, 8%)	Adopted (never Implemented) (*N*=23,21%)	Implemented (never maintained) (*N*=22, 21%)	Maintained (*N*=36, 34%)
Operational Definition		Clinics that never had contact with the New York Office of Mental Health	Clinics that had contact with the New York Office of Mental Health but never received training and technical assistance	Clinics that received training and technical assistance but never reported quarterly/monthly data to OMH	Clinics that reported quarterly/monthly data to OMH for LESS than one year since their first data submission	Clinics that reported monthly/quarterly data to OMH for at least one year.
Clinic Population	% (n)					
Clinic Type						
FQHC^a,b,c^	58.9 (63.0)	23.5 (4.0)	88.9 (8.0)	82.6 (19.0)	36.4 (8.0)	66.7 (24)
Hospital-Affiliated Clinic^a^	15.0 (16.0)	5.9 (1)	11.1 (1.0)	4.3 (1.0)	31.8 (7.0)	16.7 (6.0)
Private Practice	26.2 (28)	70.6 (12.0)	0.0 (0.0)	13.0 (3.0)	31.8 (7.0)	16.7 (6.0)
% of clinics in rural area	19.6 (22.0)	47.1 (8.0)	11.1 (1.0)	21.7 (5.0)	7.4 (2.0)	16.7 (6.0)
	Mean (std dev)					
Average number of unique annual patients	11,131.3 (30,806.0)	3,344.4 (6,355.7)	16,177.0 (30,533.1)	21,170.3 (61,484.4)	7,267.8 (6,337.6)	10315.9 (13706.5)
Average % of patients who are… (0-100)	Mean (std dev)					
women	56.5 (12.9)	55.8 (16.1)	56.9 (6.7)	56.6 (13.3)	55.8 (19.3)	57.2(6.0)
over 18 ^c^ (*n*=104)	68.7 (28.7)	40.2 (35.6)	70.6 (27.3)	75.1 (19.6)	67.8 (28.4)	78.4 (22.1)
over 65 (*n*=102)	21.5 (21.4)	26.1 (28.1)	21.6 (18.5)	19.8 (20.4)	17.4 (23.9)	22.7 (18.1)
Racial or Ethnic Minority ^a^ (*n*=102)	51.3 (35.0)	44.6 (26.4)	26.9 (34.9)	48.4 (35.1)	51.2 (33.6)	62 (36.5)
with a diagnosis of anxiety or depression ^a,b^ (*n*=102)	22.9 (21.5)	35.4 (24.7)	20.4 (12.9)	22.4 (28.4)	15.2 (20.3)	21.7 (14.5)
Payer Mix	mean (std dev)					
Commercial (Private) Insurance ^c^ (*n*=106)	29.5 (18.1)	40.3 (19.1)	35.4 (16.2)	20.7 (12.7)	34.0 (23.0)	25.6 (14.1)
Medicare only (*n*=106)	18.7 (21.5)	27.4 (32.8)	17.0 (10.8)	19.6 (20.2)	15.5 (18.1)	16.2 (19.3)
Medicaid only	47.1 (22.3)	40.6 (29.2)	46.1 (23.8)	43.5 (16.6)	54.1 (25.2)	48.3 (19.4)
Dual-eligible Medicare and Medicaid (*n*=101)	14.1 (18.8)	21.5 (31.5)	9.7 (13.1)	7.3 (6.3)	15.0 (22.4)	15.6 (14.4)
Uninsured (*n*=104)	9.1 (11.7)	8.6 (8.8)	5.2 (4.1)	10.9 (9.1)	10.5 (18.8)	8.3 (10.9)
Do you use the following for clinic reimbursement?	% (n)					
CoCm codes ^c^ (*n*=103)	54.4 (56.0)	23.5 (4.0)	25.0 (2.0)	34.8 (8.0)	89.5 (17.0)	69.4 (25.0)
Psychotherapy codes ^c^ (*n*=103)	45.6 (47.0)	5.9 (1.0)	55.6 (4.0)	65.2(15.0)	33.3 (6.0)	55.6 (20.0)
Not reimbursed at all ^c^ (*n*=103)	34.0 (35.0)	5.9 (1.0)	66.7 (6.0)	17.4 (4.0)	38.9 (7.0)	47.2 (17.0)
Average % of reimbursement (0-100)	mean (std dev)					
Psychotherapy codes ^c^ (*n*=47)	69.7 (28.8)	NA	78.4 (16.9)	54.2 (24.9)	44.7 (42.6)	87.8 (11.7)
not reimbursed (*n*=35)	15.4 (14.9)	25.0 (NA)	12.1 (18.6)	6.2 (9.2)	23.0 (24.9)	15.1 (7.6)

The Never Reached sample of clinics was significantly more challenging to recruit than clinics at the other RE-AIM steps, for which we had more detailed contact information. The first attempted contact with Never Reached clinics was via their clinic email and/or an online "contact us" form, attempting twice to contact them using these methods. Clinics without an online presence were contacted via telephone call. This was repeated a maximum of four times before marking the clinic as "unable to be contacted”. Surveys were only sent to clinics with confirmed contact information. In instances where multiple clinics reported to the same person, we asked that individual to fill out one survey for each clinic, delegate the survey to a more local leader for each clinic, or some combination of the two. Individuals completing the survey were sent a $20 gift card and entered into a raffle for a $500 gift card.

### Survey data analysis

Survey data were first examined descriptively across the clinics at each RE-AIM step. For demographic, clinical, and financial data, analyses of variance (ANOVA) were employed for continuous variables, and Chi-squares were utilized for categorical variables. In the event of a significant ANOVA or Chi-square among all surveyed clinics, two-group pairwise tests were conducted using Bonferroni adjustments for the *p*-values. These post hoc analyses further described group differences among clinics within the RE-AIM steps.

### Qualitative interviews

After survey completion, participants were contacted via email about participation in a semi-structured interview regarding their clinic’s experience (or lack thereof) implementing CoCM. Each interviewee was given a $20 gift card and an extra entry into the raffle for the $500 gift card upon completion of the interview. Interview guides were created based on the clinic’s RE-AIM step and included questions about barriers and facilitators to implementing CoCM. The codebook was developed a priori in tandem with the interview guide (Appendix 3). Interviews were conducted and audio-recorded via Zoom [13]. Audio recordings of the interviews were transcribed and uploaded to Dedoose [14], a qualitative data analysis computer program. The primary coder performed a quality check of the transcript prior to conducting the coding. The coding of each transcript was done by both the primary and secondary coders, and differences were reconciled to agreement to ensure inter-rater reliability.

The University of Washington IRB, using the definitions in the Code of Regulations part 46.102 for “Human Subjects” and “research” determined that our activities were not human subjects research and did not need IRB approval [15]. Consent was still obtained for all participants prior to participation in the study.

## Results

### Quantitative results

**Fig. 1:**
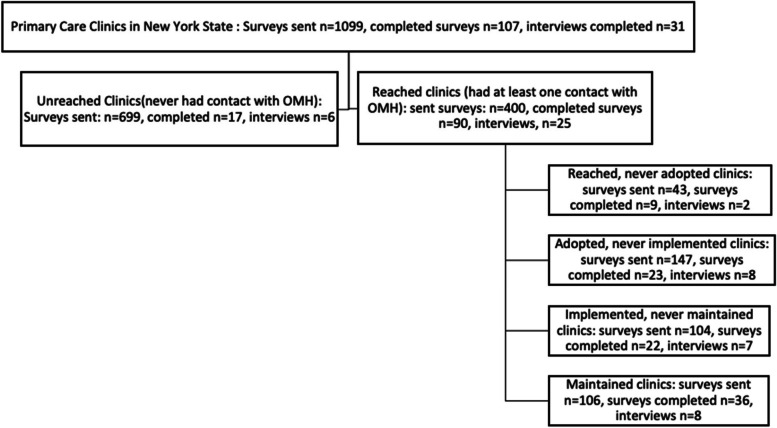
CONSORT Flowchart

A total of 1,099 surveys were sent out to primary care clinics across New York State. Of those, 107 (9.7%) were completed and included in our analyses. Further details can be found in Fig. 1. Table 1 summarizes the descriptive data collected from the surveys, organized by RE-AIM step. Recruitment efforts attempted to ensure a similar number of responses per RE-AIM step; however, the Reached step contained the fewest number of clinics (*n*=43), survey responses (*n*=9), and interviews (*n*=2). There were no significant differences among these clinics by RE-AIM step for the following clinic variables: number of annual patients, percentage of rural clinics, percentage of women patients, percentage of patients over 65 years old, percentage of patients with Medicaid, percentage of patients with Medicare, percentage of patients with dual eligible payer mix, and use of psychotherapy Current Procedural Terminology® (CPT) codes.

Significant differences were observed between Reached clinics (*n*=9) and Never Reached clinics (*n*=17), with Never Reached clinics significantly less likely to be FQHCs, (*p*=.003), and more likely to be private practices (*p*=.001). Never Reached clinics were also significantly more likely to serve a predominantly pediatric population (patients under the age of 18, *p*=.001), have a higher percentage of patients diagnosed with depression and/or anxiety (*p*<.04), and have a higher proportion of patients with private (commercial) insurance (*p*=.004) than clinics in the other RE-AIM steps.

Further differences were observed among Reached (*n*=9), Adopted, (*n*=23), Implemented, (*n*=22), and Maintained (*n*=36) clinics. Reached clinics had a significantly smaller proportion of patients that identified as a racial or ethnic minority (*p*<.036), compared to Maintained clinics, and a significantly higher proportion of BH services that were not reimbursed. Adopted clinics were significantly more likely to be FQHCs (*p*=.004) and have a significantly lower proportion of patients with private (commercial) insurance (*p*=.003), compared to Implemented clinics. Clinics in the Maintained step had a significantly higher percentage of psychotherapy codes reimbursement compared to clinics that Adopted but never implemented (*p*=.001) and Implemented but never maintained (*p*=.006)

While not reported in Table 1, analyses were run to determine any significant differences among surveyed clinics according to clinic type, rather than RE-AIM step. Hospital-affiliated clinics were significantly more likely to have used at least one CoCM billing code compared to FQHCs and private practices (chi-square *p*=.040). Private practices had a significantly higher proportion of patients under the age of 18 compared to FQHCs and hospital-affiliated clinics (ANOVA, *p*<.05). FQHCs had a significantly higher proportion of uninsured patients (ANOVA *p*<.05) and patients with Medicaid as their only insurance (ANOVA *p*<.05), compared to hospital-affiliated clinics and private practices.

### Qualitative results

We conducted 31 interviews with clinic representatives across all RE-AIM steps. Appendix 4 contains details such as interviewee job title, clinic type, and RE-AIM step. Overall, clinic representatives cited more barriers than facilitators to implementing CoCM. Clinics from all RE-AIM steps mentioned behavioral health workforce shortages and negative social determinants impacting patient care as barriers; however, these themes emerged most often in the context of accessing and/or providing BH care *in general*, not CoCM specifically. Data themes related specifically to CoCM were: CoCM billing requirements, reimbursement rates, buy-in to the model, quarterly data submission requirements, TTA, and available technology. Appendix 1 gives an evidence trace table for each domain and corresponding constructs with quotations. This paper will focus on the three most prominent themes specifically related to CoCM: 1.) Billing requirements, 2.) Reimbursement rates, and 3.) Buy-in to CoCM.

### Theme: billing requirements

Interviewees described several barriers related to CoCM billing requirements shared by both Medicaid and Medicare. Both payers presume that patients enrolled in CoCM meet the recommended cutoff scores on standard behavioral health screening tools. For example, if a patient is being treated for depression, the initial symptom screening score on the Patient Health Questionnaire-9 (PHQ-9) is presumed to be a 10 or above, as this is the current consensus on a cutoff score for diagnosing depression [16]. Both payers reimburse monthly, as opposed to each time a discrete service is provided, and both payers presume that a CoCM patient’s primary care provider is prescribing any psychotropic medication related to treatment, as opposed to a psychiatric specialist. One interviewee expressed frustration with these criteria, saying, “there's so many caveats that, somebody else can't be giving services, it has to be in certain increments, it has to meet such specific criteria…case in point, today's an admin[istrative] day. I've talked to three patients, and two of them […] don't fit the criteria” (11-M). Another interviewee specifically called out the fact that the billing occurs monthly, saying, “it stinks that they're monthly [billing] codes. I'll be very honest. I don't know that CMS [Centers for Medicare and Medicaid Services] is going to change those codes” (59-R). When describing the *differences* between Medicaid billing and other payers, an Implemented clinic reported that “one of the things that's really challenging is, there's different requirements for different payers. […] Some payers care about time spent. Medicaid doesn't. It's whether or not you had a clinical contact. The details take up so much time when figuring out the billing that it takes a lot of bandwidth on the administrative side” (17-I). “Even though we have automated billing, the [Medicaid] claims cannot go out in an automated way and they require individually one by one dropping each and every claim.[…] Nobody can quite figure out how to make this automated. Again, if you don't have somebody dedicated on the payroll to do some of this stuff, it does fall apart” (29-I).

Another difference between Medicaid and other payers is Medicaid’s retainage payment for which hospital-affiliated clinics and FQHCs are eligible. A Maintained clinic reported that the “requirements for submitting a claim for retainage are very impossible, so we have never done it. We are just taking the three quarters of the flat rate fee, if we bill at all” (50-M). Interviewees described the process for determining eligibility as "time-consuming" (17-I), with one rhetorically asking, "is it even worth it for 25%? [...] If New York State Medicaid would just pay up front the full 100% and get rid of the 25% retainage, that would make me happy" (59-R).

### Theme: reimbursement rates

Among the Implemented and Maintained clinics, NYS Medicaid reimbursement rates commonly presented perceived barriers to sustaining CoCM. Medicaid rates were described to be lower than both the alternative psychotherapy codes, and the CoCM reimbursement amounts received from Medicare and commercial plans. One interviewee said that Medicaid CoCM "detracts from our billable hours, from a financial standpoint, it just doesn't make sense. If a patient is going to meet with a therapist weekly, they would still get the same amount of therapy and we could bill $110 for the [Medicaid] Collaborative Care rate for the month, or we could bill $150 per session, per week” (08-M). Another clinic explained that “[y]ou can bill for certain things for Medicare and private insurance that you can't bill for Medicaid” (17-I). This interviewee was referring to CoCM “add-on” codes for which private practices and hospital-affiliated clinics can be reimbursed by Medicare and other payers when the CoCM minutes for the month exceed 70 minutes in the first month of service or 60 minutes in subsequent months (see Appendix 2).

Some Adopted clinics claimed that the reimbursement rate was a barrier because it wasn’t enough to justify the amount of work required to fulfill the requirements of participating in the CCMP and/or complete the Medicaid billing application. One interviewee said, "I do remember meeting with our revenue and cycle management people about the application, and going through the application. We probably did about half of it, and then they pushed back saying, ‘Why are we doing this when we can just bill for a social work visit?’ I think there [were] a lot of questions at that point in time as to why are they making us do this" (14-A).

When the reimbursement rate was considered a *facilitator*, it was in the context of expansion of reimbursable services and/or additional compensation for existing services. An FQHC at the Adopted step said they “weren't able to bill for a couple of our [BH] providers, so the Collaborative Care model stuck out to us because we could bill a little bit for those services that we were providing anyway" (43-A). An Implemented clinic remarked that almost all payers now reimburse for CoCM, so they plan to keep providing it for the foreseeable future (17-I).

### Theme: buy-in to CoCM

In Never Reached clinics, resistance to change was reported as a barrier to pursuing CoCM implementation, with one clinic saying, “people like doing what they're doing, and the implementing of change is very tough, especially for those that are older in our practice who have done what they've been doing for a long time and are not willing to change” (21-N). Clinics at the other RE-AIM steps reported CoCM buy-in to be a facilitator when it is present, and a barrier when it is lacking. One respondent said, "the number one thing that makes [Collaborative Care] work is providers understanding what services we provide and buying into it. When we have an office that just gets it, it's beautiful. It works perfectly” (17-I). Conversely, a lack of provider buy-in has the opposite effect according to interviewees. One said that the PCPs in their clinic refer patients to CoCM inappropriately, using it as a "catch-all" for anyone with a “social work need” and expecting them all to receive psychotherapy, even though this is not consistent with the model or realistic in terms of capacity (59-R). Another interviewee described the same barrier (lack of PCP buy-in) with the opposite result: too few referrals. In the case of this Adopted clinic, only eight out of eighty providers currently refer patients to CoCM (14-A).

A few respondents gave specific reasons for lack of provider buy in, citing their discomfort with prescribing psychotropic medications and/or a desire to keep medical and behavioral health in separate silos: “[p]roviders didn't particularly like [Collaborative Care] because they wanted the behavioral health team to remain fully in charge and they didn't understand [the model], [saying,] ‘Why do I need to be involved? I'm referring [the patient] to *you’*” (04-A). Another interviewee reported, “I get a lot of pushback from the primary care side saying, ‘We're not comfortable. We're not comfortable with all these medications. We don't feel like we have enough expertise. And we really think it would be better if the psychiatrist could just keep prescribing them’” (05-A). An additional respondent shared, “primary care providers were very frustrated with the external psychiatrist. There was a lot of lack of trust, […saying] ‘[W]ho is this person that I don't know, that's not responsible for my patients, who's sending me one message through the health center record around a medication that I should prescribe?’” (08-M).

Lack of buy-in from BH providers was also cited as a barrier. Brief, evidence-based behavioral interventions are a component of CoCM, (typically 30-minute sessions with a limited number of total sessions) [17], yet interviewees reported that their BHCMs were conducting 60-minute, 90-minute (59-R), and even two-hour appointments (52-I). One interviewee concluded that the pushback about shorter appointments occurred because the request came from a nurse, rather than a fellow social worker (59-R). Another respondent felt that their BHCM had difficulty with time management and wasn’t used to setting short-term goals or conducting short-term therapy. The interviewee speculated that the BHCM was of a mindset that “everybody should be seen once a week, indefinitely" (52-I), and also pointed out that this particular BHCM felt that she had a large caseload.

When interviewees cited buy-in as a *facilitator*, it was most often attributed to buy-in from organizational leadership. A Chief Executive Officer (CEO) with a background in behavioral health (05-A), a Medical Director that supports integrated BH of any kind (50-M), and an administration that values CoCM (24-M) were some of the examples given by interviewees. Buy-in was illustrated by one interviewee in the "teamwork between medical support and executive director CEO" (30-I). The success of this teamwork eventually led to increased buy-in and implementation spread to other clinics within the organization. However, in another clinic, although there was initial buy-in expressed, it was not accompanied by active support from leadership. This interviewee stated, "I think our CEO was like, ‘We should do this. It's a good way to get revenue.’ But then it was on myself and our billing director to figure it out" (08-M).

## Discussion

The quantitative survey results align with what we would expect to see regarding the patient and clinic demographics of New York State primary care clinics, based on clinic type. FQHCs serve significantly more Medicaid and uninsured patients than other clinic types. Additionally, clinics with a large Medicare population have a large population of patients ages 65 and older. In terms of BH services, the complex interplay of permissions and restrictions on the provision of behavioral health services based on clinic type (see Appendix 2) can be seen in the quantitative data. For example, hospital-affiliated clinics generally offer fewer billable BH care services than private practices and FQHCs. Survey data indicated that hospital-affiliated clinics were significantly more likely to bill CoCM codes than the other clinic types, possibly because of these limitations. Since CoCM is billed as a primary care service, rather than a BH service, the aforementioned limitations do not apply, giving hospital-affiliated clinics the ability to offer behavioral healthcare to more patients.

Qualitative data illuminated a few important facilitators: (1) having non-clinical staff devoted to performing tasks related to Medicaid billing and data reporting, and (2) the importance of buy-in from primary care providers and organization leadership. Interviewees across clinics agree that Medicaid billing and data reporting are time-consuming activities, and having adequate, dedicated staff members to perform these tasks was consistently seen as a facilitator. While some clinics reported utilizing staff to perform these tasks on an ongoing basis, others discussed working with their report writers, data analysts, and/or billing staff early on in implementation to build electronic reports or processes to automate some tasks, underscoring the importance of cross-clinic collaboration to streamline implementation workflows.

The other major facilitator was buy-in from primary care providers and organizational leadership, which previous research has shown are key components of a successful CoCM implementation [18]. Interviewees in this study described provider buy-in as understanding the model and leadership buy-in as valuing behavioral health care. Some of the data indicated, however, that leadership buy-in also needed to include active involvement in the roll out of CoCM to be viewed as a facilitator.

The largest barriers to CoCM implementation for New York State Medicaid billing, according to the qualitative data, were: a difficulty meeting the billing requirements for both Medicaid and other payers, the low Medicaid reimbursement rate compared to other payers or fee-for-service psychotherapy, and a lack of buy-in to the model by care providers. These themes also appeared to be interconnected, with interviewees often citing one barrier as the reason for another.

The monthly Medicaid case rate billing requirements are complex and were established prior to the introduction of the time-based Medic*are* requirements in 2018. While the addition of Medicare and commercial payer reimbursement benefited practices financially, interview data suggests it also led to frustration among practices attempting to bill across different payers. In August 2018, OMH updated the Medicaid billing to more closely align with Medicare with the intention of streamlining billing across payers. Clinics can now choose to bill the minutes-based CPT codes (like Medicare) when the time requirements are met or can continue to use the original case management procedure code when the NYS Medicaid requirements are met but they have not met the minutes criteria. Even so, the NYS Medicaid requirements must be met regardless of which code is used, and Electronic Medical Record software is not designed to track services in this way. Clinics had to build reports, program new EHR functionality, and/or have dedicated staff manually track and verify criteria in order to bill.

Interviewees citing reimbursement rate as a barrier tended to refer to the Medicaid case rate amount *without* the 25% retainage, even though their clinics were eligible to receive it. With the retainage, Medicaid reimbursement rates exceed Medicare rates (See Appendix 2). In addition, Medicaid billing requirements are process-based, rather than minutes-based, so eligible clinics can feasibly receive $150 per CoCM patient per month even if they are spending *less* than 70 or 60 minutes on care. Moreover, psychotherapy codes can be billed along with CoCM codes across payers, as long services don’t overlap. At least one interviewee did not appear to be aware of this, saying that it was an ‘either/or’ proposition that didn’t make financial sense (08-M). While it may be true for the interviewed clinics that the reimbursement rate does not justify the amount of work involved in tracking and meeting the billing requirements, these and other clinics might benefit from reevaluating their billing workflows. It would be prudent to consider the aforementioned nuances and explore alternative ways to streamline processes and maximize reimbursement.

When interviewees cited lack of buy-in from primary care providers as a barrier, it was demonstrated through inappropriate referrals, discomfort prescribing psychotropic medications, distrust of the psychiatric consultant, a preference for siloed primary and behavioral health care, a preference for traditional psychotherapy over brief, evidence-based behavioral interventions, and not understanding the model. A clinic cannot provide or bill for Collaborative Care if the providers are referring patients for services other than BH care and are not willing to prescribe psychiatric medications or work with the other members of the team. When BHCMs cannot or will not provide brief, evidence-based behavioral interventions in favor of longer, weekly psychotherapy sessions with patients, this demonstrates a lack of fidelity to the model and the clinic should not expect to get the same patient improvement outcomes associated with CoCM [19]. Therefore, it would be reasonable for future improvement efforts to focus on increasing understanding of the model among all providers and training in brief, evidence-based behavioral interventions for BHCMs.

## Limitations

While this study repeatedly attempted to elicit an equal amount of responses from clinics at all of our defined RE-AIM steps, we were unable to recruit enough Reached clinics in order to determine statistically significant differences between this step and the other RE-AIM steps. We also lacked adequate representation of rural clinics. An additional limitation to this study is that the survey and interview questions were retrospective. Respondents received the survey in 2021, but were asked to complete it for the year 2019. The same instructions were given for the qualitative interviews, but it is possible that this self-reported data was not representative of 2019. This is evidenced by most participants mentioning the COVID-19 pandemic as a barrier to implementing CoCM and/or to providing care in general at some point during their interviews. In addition, the employees completing the survey and interview may not have been involved in their clinic’s initial implementation of CoCM. Furthermore, categorization of clinic RE-AIM step was based on OMH’s 2019 data. Quantitative and qualitative data for this study was gathered in 2021 and 2022, and it is possible that some of the clinics had progressed onto the next RE-AIM step or ceased implementing CoCM altogether during this time. Qualitative interviews were conducted using the interview guide that aligned with a clinic’s 2019 RE-AIM step, however self-reported quantitative and qualitative data may have been impacted by the time discrepancy.

## Conclusion

Data indicate that facilitators to CoCM implementation for New York State Medicaid billing are having dedicated staff to manage billing and data reporting and buy-in to the model from primary care providers and organizational leadership. Barriers include: difficulty meeting the billing requirements for both Medicaid and other payers, the low Medicaid reimbursement rate compared to other payers or fee-for-service psychotherapy, and a lack of buy-in to the model by care providers.. These perceived barriers may reflect infidelity to the Collaborative Care model and/or inadequate billing workflows. Apart from more education, training and planning around Collaborative Care best practices and financial sustainment, future efforts might also focus on the development of electronic medical record and billing workflow technologies with the purpose of supporting CoCM billing and best practices.

### Supplementary Information

**Supplementary Material 1.** 

## Data Availability

The datasets used and/or analyzed during the current study are available from the corresponding author on reasonable request.

## Abbreviations

ACA: Affordable Care Act
ANOVA: Analysis of variance
BH: Behavioral Health
BHCM: Behavioral Health Care Manager
CCMP: Collaborative Care Medicaid Program
CoCM: Collaborative Care Model
CPT: Current Procedural Terminology®
FQHC: Federally Qualified Health Center
NYS: New York State
NYSOMH: New York State Office of Mental Health
RE-AIM: Reach, Effectiveness, Adoption, Implementation, Maintenance
REDCap: Research Electronic Data Capture
TTA: Training and Technical Assistance
