# Supplementary material for: Barriers and facilitators to collaborative care implementation within the New York State Collaborative Care Medicaid Program
Source: BMC Health Serv Res. 2024 Apr 24;24:505. doi: 10.1186/s12913-024-10909-0 (PMC11040977; doi:10.1186/s12913-024-10909-0)
Supplement: Supplementary file 1 — Supplementary Material 1. [file 12913_2024_10909_MOESM1_ESM.docx]

Appendix 1: Selected qualitative thematic evidence trace table

| **Theme** | **Facilitator** | **Barrier** |
| --- | --- | --- |
| **Behavioral Health Workforce: Shortage of Providers** |  | "I don't know if we are staffed at this point to offer [behavioral health services]. I don't know, I guess we would have to hire extra staff, because we're pretty busy." 41-N |
|  |  |  |
|  |  | "A lot of psychiatrists or even behavioral health providers or whatever are leaving their in-person jobs and then going to consulting firms basically...it's complicated because in theory, that should expand mental health access. But then it's also like they're leaving clinics in the lurch." 32-N |
|  |  |  |
|  |  | "We have a 50% shortage of social workers. We have a 50% shortage of medical providers. Yet at the same time, we are now noticing that we have 1000 times the need that we had before." 16-A |
|  |  |  |
|  |  | "The collaborative approach can be very difficult for two reasons. One, retention of social workers. They are like gold dust. No seriously. And the hardest part has just been about keeping the social workers." 04-A |
|  |  |  |
|  |  | "There's been a lot of people leaving the profession right now, just from burnout and feeling tired, and we are not immune to that here either." 30-I |
|  |  |  |
|  |  | "We struggled just finding BHS staff. I think staffing is just one of those things that we have ebbed and flowed. Sometimes staffing's been great, and then other times, we had two people leave at once, or someone started and was getting trained and then they left. Staffing, I think, can make or break your success in the program. Because obviously you can have all of the workflows ready to go, and you can have primary care doctors that are willing to do things, but if you don't have BHS staff to see patients, it's not going to work." 31-I |
|  |  |  |
|  |  | "There is a real crisis in being able to get social workers to work in hospitals. I don't know if that is out west as well, but we have had a very, very hard time keeping them on because they can work from home somewhere else, not be exposed to anything and not have to commute, it is very unfortunate. It makes me worried about the future of hospital social work, frankly. We have just lost so many people and I know that they are now working from home. Part of it is COVID, and part of it is just the advent of telehealth...and the social workers get dumped on traditionally in medical settings. It is just a fact and they are tired of it, and they want to just do psychotherapy. And you can quote me on that." 50-M |
|  |  |  |
|  |  | "I've got 40 PCPs, I need 10 behavioral health care managers. Two and a half doesn't cut it. It just doesn't cut it, and it then makes it look like it's not working, even though it's working. We're just not staffed appropriately." 11-M |
| **Billing: Requirements** |  | "I understand the reasons why we have these protections [requirements to bill] in place, but they're barriers. It's an unintended consequence of not allowing more billable services embedded in primary care and not having more access under Article 28 and really funneling everything through OMH. There are so many guidelines with Article 31. The building guidelines and just the windows and everything, it's so prohibitive, unfortunately. And I know why; it's to protect our patients, but in our area we've discovered it's a barrier. I would say that we saw the benefit of it, but it's also the only real option we had for embedding billable services." 59-R |
|  |  |  |
|  |  | "I do remember meeting with our revenue and cycle management people about the application, and going through the application. We probably did about half of it, and then they pushed back saying, "why are we doing this when we can just bill for a social work visit". I think there was a lot of questions at that point in time as to why are they making us do this." 14-A |
|  |  |  |
|  |  | "One of the things that's really challenging is, there's different requirements for different payers. You can bill for certain things for Medicare and private insurance that you can't bill for Medicaid. The details take up so much time when figuring out the billing that it takes a lot of bandwidth on the administrative side. That's definitely a challenge." 17-I |
|  |  |  |
|  |  | "It's not easy. I think we've just accepted the fact that billing Medicaid is typically not easy. It is what it is. But what becomes challenging is not just billing the monthly rate to Medicaid, it's the retainage piece that becomes very challenging and time consuming." 17-I |
|  |  |  |
|  |  | "There were issues with billing acceptance that we did not submit it correctly. And then a whole bunch of things that should have been reimbursed were not." 52-I |
|  |  |  |
|  |  | "Our billing system is very complicated and no one understands it, except for the people in billing. So the billing has been the biggest challenge. The requirements for submitting a claim for retainage are very impossible, so we have never done it. We are just taking the three quarters of the flat rate fee, if we bill at all. It is a lot of work for not a whole lot of money." 50-M |
|  |  |  |
|  |  | "First of all, there are so many caveats: somebody else can't be giving services, it has to be in certain increments, it has to meet such specific criteria...even when you think you're doing everything right down to the T, you'll still get a denial. So, that's frustrating." 11-M  “Even though we have automated billing, the OMH claims cannot go out in an automated way and they require individually one by one dropping each and every claim.[…] Nobody can quite figure out how to make this automated. Again, if you don't have somebody dedicated on the payroll to do some of this stuff, it does fall apart. That's the big thing I hear is that people just don't have somebody in the capacity and role and skillset that [our Billing Lead has to do this and then it falls on the director of the program or the clinicians or the billing staff. They don't understand it. They come back denied or so you're in this circle of difficulty that is hard to sort out. So then people get frustrated and say, forget it, this is just not worth my time. It's costing me more than I'm actually getting reimbursed.” 29-I |
| **Reimbursement Rate** | "We did Collaborative Care back in 2019. Staffing looked very different. We weren't able to bill for a couple of our providers so the Collaborative Care model stuck out to us because we could bill a little bit for those services that we were providing anyway." 43-A | "The reimbursement rates are really kind of the hardest thing for us, especially with the Medicaid population. I mean, those are typically the individuals who need it, and the reimbursement is not there to actually drive a profitable program and at least stay in the black." 38-N |
|  |  | "The Medicaid routine is just a nightmare...it's just like, is it even worth it for 25%? I don't know. It stinks that they're monthly codes. I'll be very honest. I don't know that CMS is going to change those codes. If New York State Medicaid would just pay up front the full 100% and get rid of the 25% retainage, that would make me happy." 59-R |
|  |  |  |
|  |  | "I'll say the reimbursement or the compensation didn't make sense from the extra amount of work that was being done." 03-A |
|  |  |  |
|  |  | "[Filling out a billing application] just didn't really work for us because what we were being paid and what we were paying out wasn't tying together...that's why we have a new social worker team now. We can now bill directly for the social worker team, which means that we'll recoup our investments." 04-A |
|  |  |  |
|  | "… having so many payers now reimbursing, almost all of them reimburse for it. I don't see why we would stop." 17-I | "I don't know why they reduce the amount of Collaborative Care [reimbursement] after a year…The children still have the condition after one year...to reduce it down to $75 from $112? Why? The children still need the service." 06-M |
|  |  |  |
|  |  | "It [Medicaid] detracts from our billable hours, from a financial standpoint, it just doesn't make sense. If a patient is going to meet with a therapist weekly, they would still get the same amount of therapy and we could bill $110 for the [Medicaid] Collaborative Care rate for the month, or we could bill $150 per session, per week. Financial aid, it doesn't really make a good business case for us." 08-M |
| **Buy-In: Providers** | "I would say the majority of the PCPs find it valuable. They've also supported, "Yes, we want this. Yes, we want to have somebody we can talk to and ask questions to." It's coming from the provider, but it's also coming from the top to say, "Yes, the supports are for the primary care docs as well."" 03-A | "There's a lack of ability to change in our workers. People like doing what they're doing, and the implementing of change is very tough, especially for those that are older in our practice who have done what they've been doing for a long time and are not willing to change." 21-N |
|  |  |  |
|  | "The providers were really encouraged to refer and encouraged to know more about the Collaborative Care program. The office that we utilized Collaborative Care in, the providers all knew what was going on, they knew how to get the patient seen, and they did all of the warm handoffs with that provider. I think that helped a lot." 43-A | "Collaborative care here has become, "Okay, this patient has this social work need. I'm sending them every patient I possibly can." And our primary care providers really want that psychotherapy. They are really looking to get that psychotherapy in primary care. They refer just about every patient for that. They want to give counseling and therapy within the collaborative care model, and that's not how it's necessarily supposed to be. Yes, there's a component of it, but it's meant to be the smaller portion... I want to word that carefully, but collaborative care seems to be a catchall. And providers, the primary care providers, we do reeducation and we do reminders, but it's hard to tell them that we can't help these patients, because they don't fall into this box of collaborative care model." 59-R |
|  |  |  |
|  | "The number one thing that makes it work is providers understanding what services we provide and buying into it. When we have an office that just gets it, it's beautiful. It works perfectly." 17-I | "I definitely think we don't have full buy-in. We have 80 primary care providers here, and we have 8 that are consistently training and trying to stay abreast of behavioral health issues, so that's not a very large number considering the grand total of people. So, we'd love to see more buy-in from the provider level but I'm not sure we always get that. I think we had, at that point in time, what I would consider about 35 internists or family medicine doctors who were seeing patients that would be eligible. We had 13 participating. That number has since dwindled to 8, so we did not get to 50%, so I can't even say 50/50." 14-A |
|  |  |  |
|  | "It's having the combination of primary care support and dedicated Collaborative Care clinicians." 30-I | "I think the biggest challenge is just providers understanding what Collaborative Care is. It's something that sometimes when you're doing the work, it's hard to fully grasp it. It's so different and so flexible, which is amazing, but also because of that flexibility, it's hard to be able to give someone a black and white, like we do this and we don't do that." 17-I |
|  |  |  |
|  | "I think we have clinical buy-in for sure." 26-M | "I think a barrier for us, specifically for behavioral health care management, is really provider understanding and buy-in of what it is versus what it isn't. At the end of the day, they want the health and the good outcomes for their patient and they want it to run smoothly. What they don't quite get or are interested in accepting sometimes is the parameters." 11-M |
|  |  |  |
|  |  | "I have requested that our behavioral healthcare managers do an expert screen on all new Collaborative Care patients and referrals, so they have implemented that this year and are doing that. They weren't doing it on everybody; they were doing it as like, "Oh, I've identified this might be a need." And I said, "No, no, we do it on all new patients." I'll be honest, our behavioral healthcare managers were doing 90-minute appointments and hour-long appointments. Collaborative Care is not supposed to do that. I'm a nurse by trade, and a nurse telling social workers, "You don't need to do an hour-long appointment. You don't need to do 90 minutes." didn't go over so well." 59-R |
|  |  |  |
|  |  | "Our previous social worker, there were times where she was spending too long with individual patients. So she would be in there for like two hours with them and then her caseload was getting... She felt like it was too heavy, but I think it was the time management that was hard for her. And she also had difficulty with kind of the short-term goal, like kind of short-term therapy and figuring out how to kind of facilitate therapy in that way, rather than thinking that everybody should be seen once a week indefinitely kind of thing." 52-I |
| **Buy-In: Organizational Support** | "I believe it was my CEO [who pushed for CoCM], and who happened to be a social worker by training. He has always had an eye towards behavioral health just because of his training." 05-A | "I'm not sure that the complete organization truly understands the importance of behavioral health when it comes to assessing someone's overall wellbeing." 14-A |
|  |  |  |
|  | "It has been a top down support from the very beginning, day one." 03-A | "I think our CEO was like, "We should do this. It's a good way to get revenue." But then it was on myself and our billing director to figure it out. And I think it ended up being pretty frustrating for the therapist because it was just kind of an extra burden for them to have to carry out...is this Collaborative Care, is this therapy? And it was a whole different set of requirements. So I think we got some resistance on the people carrying out the program as well." 08-M |
|  |  |  |
|  | "The teamwork between medical support and our executive director…Our CEO is on board with this program. It has a lot of support behind it from several departments as well. [Providers] knowing that they can refer people into this program, as long as they meet these criteria, has been really beneficial and helpful." 30-I |  |
|  |  |  |
|  | "I think the program is viewed as very valuable by the administration." 24-M |  |
|  |  |  |
|  | "Our medical director for primary care, who hired me actually, has always been very supportive of integrating behavioral health in whatever way possible." 50-M |  |
| **Data Submission: Reporting data** | "We really worked with our report writers to try to get the report so we didn't have to pull some of this stuff manually, so that was helpful." 31-I | "Then for the quarterly reporting, that's me. I worked with our report writer here and she was able to create a report on a spreadsheet that we were previously able just to submit, which worked out really nicely. It's not hard by any means, it's just time consuming when we have that many clinics to report for. It worked easier for us when we could just submit the spreadsheet, but I know on previous data calls, they said that the spreadsheet wasn't working. Maybe somebody was doing something wrong, or the fields weren't filled in correctly. That's been a new development over the past two quarters. It's just time consuming." 31-I |
|  |  |  |
|  |  | "The data has been the bane of my existence for the last five years...[the data] is only as good as it is inputted by the staff. That's definitely a big barrier, but also, we've run into challenges where the reports that we built are pulling data from our EMR. If the EMR is upgraded and then something breaks, we don't notice it until the next quarter when we're pulling data." 17-I |
|  |  |  |
|  |  | "I feel like the questions are confusing, like what data for the numerator, what data for the denominator? Specifically for the depression and anxiety screening yield, it was like, "Oh my God." And we'd have to run the numbers like 10 million times to make sure that we were actually measuring what was actually needed. And so I think that was a really big challenge in the first year. It would take us either a whole day or a few days within the week of like, "Okay, sit down, go through this information, is this the..." So it was so much. So I think that was one thing." 07-I |
| **Data Submission: Having a Data-Dedicated Staff Member** | "I think it helped that we had people...to help run the reports and data. So I think it made it easier for us." 10-A | "I think the big challenge of the data is making sure that the care managers have their spreadsheets up to date. And now that we are moving into this automated version, I'm hoping that problem will be less of a problem." 50-M |
|  |  |  |
|  | "We are blessed to have [someone] who manually enters everything and supports [data entry] alongside the clinicians. She helps send out reminders. She keeps track of the data outcome. She keeps track of who needs what. The clinicians are just way too overloaded and way too busy to do that on their own." 29-I |  |
|  |  |  |
|  | "I think using our data analysts. The fact that we are a larger system and that we have staff available with that expertise was really helpful." 17-I |  |
|  |  |  |
|  | "I submit the data. So I've been part of the program for a few years. I...help certainly." 24-M |  |
|  |  |  |
|  | "Our population health department has a data team that has been on the project working with me and with the social work manager to create this automated registry and to make changes within our EMR to make the screenings easier to do and integrated within the electronic medical record." 50-M |  |
| **Social Determinants Impacting Patient Care: Complex Mental Health Conditions** | "Interestingly enough, for patients with Medicaid with straightforward depression and anxiety, I would say Collaborative Care has been great and has really streamlined our mental health care for those patients." 47-M | "I feel like the demand has gotten so complex and so intense that it's getting harder for the primary care folks to carry the complexity along with everything else." 32-N |
|  |  |  |
|  |  | "A lot of our patients actually start out feeling suicidal and we may even have to refer them to psychiatric hospitalization. To have a PCP manage so many patients and actually deal with a psychiatric crisis, a lot of the time, the patient is not stable and has to change their medication every week. This is just too much for the primary care doctors. Unless they actually have experience with more severe diagnoses…it could actually scare them." 67-R |
|  |  |  |
|  |  | "[We serve] a very high needs population, specifically at this one practice. So, I think that there is a struggle with limited resources. I'll often get referrals for patients that aren't necessarily the best candidates for CoCM. So we have a lot of acute patients with schizophrenia or bipolar that aren't necessarily engaged with treatment in the community. So there is a high need for that [and] I think the challenges are just the high needs. And in terms of social needs, I've tried to really help people manage the stress because I don't necessarily think we're going to resolve all of the social needs they have. I have a lot of patients that have a history of substance abuse, unstable housing, unstable financial support, whether it be employment or SNAP benefits, so I just think that in terms of the needs of the population kind of obviously interferes, or is a reason, for their increased depression or anxiety." 10-A |
|  |  |  |
|  |  | "Right now there's such a mental health need in crisis, there are a lot of new patients coming in. So then to just be able to help make sure everybody gets what they need and what they're looking for can be a challenge too." 30-I |
|  |  |  |
|  |  | "I think in the peak of COVID, some people were just at their wits end. It was kind of almost like things were erupting for people that was like, "I didn't even know that I had these issues going on." And with everything going on with COVID, the system is very, very saturated. And so for those patients who were having acute symptoms, it was hard...like hospitals were full." 07-I |
|  |  |  |
|  |  | "Depression and anxiety is unfortunately, really, really prevalent in our patient population." 24-M |
| **Social Determinants Impacting Patient Care: Language and Literacy** |  | "And there's also a language barrier. It's primarily Spanish. I think that is, primarily, our biggest barrier." 21-N |
|  |  |  |
|  |  | "A lot of our patients are not fluent in English. So if they are not getting the mental health services here, they're not getting the mental health service anywhere." 67-R |
|  |  |  |
|  |  | "We realized patients couldn't read it. They needed help. It wasn't the language. It was the literacy. So it made us...really understand the needs of our patient population I think more than we probably would've on our own." 05-A |
|  |  |  |
|  |  | "I think one [challenge] is language barrier, and cultural stigma is definitely something that we have to overcome all the time. I think more than 40% of our adult caregivers, don't speak, and can't read or write in English. We have a very diverse population. And so that communication, if that's being done via an interpreter, can sometimes be a barrier as well as cultural stigma. I have some parents who just don't really buy into mental health care." 52-I |
|  |  |  |
|  |  | "I would say language access and education has been a big issue for our refugee population. Some of them have no former access to healthcare. Sometimes stigma and conversations around mental health in their home countries…it's been a challenge. There's not a ton of literature on the populations we serve [relating to their] mental health services." 08-M |
|  |  |  |
|  |  | "We have a lot of patients with low health literacy and so, because the providers don't really understand and don't have the buy-in, it's hard to explain the concept of CoCM to the patient who already doesn't have that health literacy." 11-M |
| **Social Determinants Impacting Patient Care: Patient Engagement** |  | "And just, in regards to following up in counseling, no one seems to...they no show appointments and then they come back to us saying, "Well, let's just stay on this med because it's easy," even though it's not working." 21-N |
|  |  |  |
|  |  | "We struggle to engage them via telephone and virtual visits, between primary care visits. Many times their phones get turned off or their minutes are up or they don't have internet access. We do struggle with that specifically, in between appointments." 59-R |
|  |  |  |
|  |  | "I think the non-engagement of patients [has been the biggest challenge]. [There's people] who have stopped answering their phone for whatever reason, people who've stopped engaging with the providers, have stopped coming to appointments, missed appointments, people who aren't necessarily, like I said, doing the work. So, they stop making goals, and stop trying to develop coping skills. And also, I think for me, it's been really hard and challenging to get people engaged and kind of work on those things when they have so many other problems and challenges in their life. If they're in a neighborhood where there are shootings every night and they're afraid to step outside because they're unsafe and there's just a lot of things going on, maybe their utilities are being turned off or they don't have enough food, it's hard for them to kind of focus on trying to come up with coping skills when they have kind of this chaos that they live in." 10-A |
|  |  |  |
|  |  | "Sometimes our population doesn't actually believe in therapy either. So what they're doing, they come in in a states of crisis, we deal with whatever the primary modality is, then we don't see them again. It doesn't mean that the problems are solved, it's that suddenly, "Well my husband's returned. So I don't need to speak to you anymore." 04-A |
|  |  |  |
|  |  | "I think another challenge is getting some of the patients engaged fully with us because they'll enroll and then they'll just drop off. You won't hear from them. The engaging part is always a challenge. I think there's a lot of disengaged patients. For whatever reason. It's hard." 23-I |
|  |  |  |
|  |  | "Having a very highly stressed patient population. I mean, it's challenging to keep people in care long enough to affect change, right? That's probably the biggest barrier, to keep people in care long enough to reduce symptoms. They drop out of all contact...our patients are very transient. If their cell phone numbers change, because they often have prepaid, so numbers will change, the addresses will change and they just disappear for a while. I think that's probably the most difficult piece. Sometimes 70 days feels like a lifetime. For other people, it feels like a blink of an eye." 29-I |
|  |  |  |
|  |  | "There are many challenges. Show rate is very low within the Medicaid world. People are socialized not to cancel their appointments when they can't come, and also, there are many psychosocial factors that get in the way of people being able to make their appointments." 50-M |
|  |  |  |
|  |  | "I think there's always some adherence with appointment problems, which is sort of both easier and harder with COVID. It's sort of easier to make a video visit...but they may be less engaged having never met the manager in person." 24-M |
| **Social Determinants Impacting Patient Care: Availability and Accessibility of Higher-Level Behavioral Health Care** |  | "And so then when you try to refer out, you're met with resistance and barriers. Whether that be that there's just not enough resources or they can get there, or X, Y, Z...a lot of places are not taking new patients. So I know a lot of our medical board, we've talked over the years how great it would be to implement behavioral health into our clinic, day to day." 32-N |
|  |  |  |
|  |  | "It's just really hard to find a place that would take kids as well. I think that was mainly the issue. Because a lot of [specialty mental health places] would only take adults. So just finding pediatric specific or family counsel for teens and adolescents, that was really the only barrier." 27-N |
|  |  |  |
|  |  | "I'll be completely transparent, there aren't enough resources for behavioral health in our area and it's hard to get into places. And then even some places that people can get into, sometimes finances are limiting, because they have to be able to afford it." 59-R |
|  |  |  |
|  |  | "I'll tell you, they [the mental health clinic they refer out to] lost their psychiatrist, and he is the only one in 60 miles. And then he was the supervising or collaborating physician for all the rest. They're not taking any new patients." 64-A |
|  |  |  |
|  |  | "We have an outpatient mental health clinic attached to our hospital, but unfortunately they cannot handle the volume of referrals that we get. And they only take certain insurances, so we do refer to our two county mental health agencies a lot of the time. But they're often full or not taking new patients or have a long wait list." 31-I |
|  |  |  |
|  |  | "We got to a point where Samaritan was like, "Hey, can you stop sending those patients? Because we just don't have the capacity". I kept trying to push as many as I could. But there was a point where the intake person was kind of like, "We literally don't have any...we're just too far out". Yeah. It's been a rough ride." 07-I |
|  |  |  |
|  |  | "We're in an underserved area, our providers will see behavioral health issues and to the extent that they can manage it, they will, with medications. But then there might come that point in time when it is beyond them and we need to refer out to a psychologist, social worker, or a psychiatrist. Where the problem comes in our area, is just getting some kind of service for those patients that need help. It's very difficult to get appointments. It's a problem. It's a big problem." 01-M |
|  |  |  |
|  |  | "Because we are so rural, even if that patient needs a higher level of care, it might be months before they get it." 11-M |
| **Social Determinants Impacting Patient Care: Transportation** |  | "We are in a low socioeconomic area with very limited public transportation, so anytime that we have an individual who's in need of going to these outward facilities, transportation becomes an issue. We have Medicaid transport that we can use and we have GetThere, but a lot of times, especially with the COVID pandemic that we have going on, those opportunities have dwindled very quickly in order to be able to transfer these people and their needs." 13-N |
|  |  |  |
|  |  | "Transportation in the area has gotten really terrible lately. We have had a hard time getting people to appointments and we've had Medicaid cabs refusing to transport patients because they have bedbugs. GetThere has been a great resource for us and we've used that for a while, but then now everything needs to be approved by their manager. We're seeing a decrease in the ability of GetThere to do the transportation for our patients and they want us to try to coordinate through the insurances more. With Medicaid, they really don't do same-day transportation and you got to coordinate ahead of time and it's time consuming and it's a burden." 59-R |
|  |  |  |
|  |  | "I think barriers to care in general, like transportation, lack of follow up sometimes with like phones, people don't have minutes on their phones, things like that. We utilize a lot of our case management services to address a lot of these barriers to care. So they assist with those patients that might be higher needs, that may need more of an assistance. They may need more assistance in getting to the health center and getting to other services. We see a lot of farm worker patients as well. So, them moving around a lot and things like that, sometimes it gets hard to track patients. But, we try to make sure that our patients know how to reach us when they're ready." 43-A |
|  |  |  |
|  |  | "Socioeconomic barriers. So can our patient get to us? If they don't let us know that they can't get to us, then we don't know how to help. So our patients, do they have transportation? Do they have food? Do they have childcare? Do they have different things like these? Because their social challenges are barriers to their physical health and their mental health. And so for our community, we have to make sure that their basic needs are getting met so that they're able to effectively give themselves to the treatment plan." 07-I |
| **Technology: Electronic Medical Records (EMR)** | "Having more efficiencies with our EMR and more automated reports has just made it [CoCM] easier." 17-I | "… trying to adapt with everchanging electronic medical records, screening things, and PCMH requirements." 32-N |
|  |  |  |
|  | "Our EMR makes communication really easy so that whenever we discuss a patient, we include the PCP. So I think that has been helpful." 47-M | "The only thing [challenge] was when we moved to the new EMR, we had a little bit of a snag trying to come up with our workflow for Collaborative Care in the new system, because it didn't necessarily have anything built in for Collaborative Care, per se. So we had to work really closely with IT to get reports built in for our billing. It wasn't necessarily made for collaborative care, per se. So we've kind of had to work with them closely to build in all of those things. So that was probably the only snag, was when we went live in May, working with the individuals to get the reports built into the system." 10-A |
|  |  |  |
|  |  | "Sometimes the communication that needs to happen in Collaborative Care is either delayed or doesn't happen or happens in a way that there are so many forums for communication and people might be used to having verbal communication, but then they get a secure health message [in the EMR] and they never check it." 50-M |
| **Technology: Telehealth** | "Its kind of hard not to do a telehealth appointment. You have to either not have the technical ability, or you just really don't want to come to your appointment. It's not that hard to hit accept on the Zoom meeting. It's just not hard. I think we've probably actually been able to be a little bit more helpful for some of our more distant locations because of telehealth. Instead of a patient having to drive two hours for an in-person eval[uation], they can just do it whenever. That's probably been helpful. So less no-shows, and probably a little bit of a bigger reach." 03-A | "We keep saying, "Oh, this is going to increase access with the pushing of Telemed," but it almost makes the divide bigger for some subgroups, like the poor elderly folks who can't figure out how to make a Zoom. Or for some of my more poverty stricken families who don't have internet at home, or they're on a phone, and if they got a good cell tower, then you're good to go. But otherwise, yeah, it's definitely a conundrum." 32-N |
|  |  |  |
|  |  | "[The benefit of] telemedicine depends, because our patient population is typically more geriatric than the average, especially with our primary business. So getting them to utilize telemedicine, that's always a hurdle. The reliability of them actually showing up to that appointment, it's not them coming in and coming into a clinic setting, we just expect them to pop on at nine o'clock...getting them to actually do that has been a hurdle. But we're trying to work through a couple different things, whether it's we can equip them with some telemedicine devices, chronic care management, remote patient monitoring, and other things that would make [telemedicine] a little bit easier for them...or appointment reminders, or anything like that the day before or that morning, little things like that to try to kind of streamline it." 38-N |
|  |  |  |
|  |  | "We also have a number of patients who are anxious about leaving their homes. With that, then we couple the fact that patients don't have the equipment that they need to really totally be able to receive the full services. Either they have spotty Wi-Fi, or they just don't have the equipment." 16-A |
|  |  |  |
|  |  | "So during COVID, the lack of internet. Elderly people who are not tech savvy, and even our own staff didn't want to set up visits. It's such a drastic change for them. And our patients, they wanted to see their provider regardless of choice. But we're heavily weighted on the 65 and older." 64-A |
|  |  |  |
|  |  | "When we were onsite, it was a little different because in the telehealth world, it's hard to go into a hallway, like "Can I grab you real quick and talk about this question I have with medical". It's not impossible, it's just like you have to seek it out a bit more overtly." 30-I |
|  |  |  |
|  |  | "We live in fairly rural counties. So sometimes we struggle with good internet connection and data concerns being an issue for a patient. And just good connections in general." 31-I |
|  |  |  |
|  |  | "Now, in the age of telehealth, there are all kinds of connectivity issues or people not having devices because they can't afford them or their phones are turned off. So even those visits that are scheduled for telehealth often don't happen. So sometimes we are actually doing visits just over the phone, which in psychiatry, is somewhat acceptable, but in other specialties it's awful. That is one challenge." 50-M |
| **Technology: Patient Tracking Registry** | "We're constantly using CMTS to show where we're at." 29-I | "It was more of a challenge on my end to do that registry and to keep track of so much that was happening. I remember that being the hardest for me, to run the registry and group everything together and everything like that, because I really just wanted the provider to see the patients. It was a lot of tracking." 43-A |
|  |  |  |
|  | "We use a registry, so it's super easy for me to look at it, as long as my behavioral health care managers are getting those names in that registry. It's super easy to look at and be able to help again with that practice transformation." 11-M | "The first year, I think it was the understanding of the CMTS program and what kind of things I could put in and...all the information that needed to be within the system in order to properly measure all the things that we were doing with the patient. And then learning about the additional leading questions, like the depression screening rate, the depression screening yield, and the generalized anxiety screening rate and yield. Those are so difficult, they would take us all day to try to figure out with our EHR, we were trying to say, "What do we measure?"" 07-I |
|  |  |  |
|  |  | "We're actually switching to the registry with the University of Washington. We're having our training meeting with Ashley today. So up until this point, the depression care managers have each just had their own Excel spreadsheet. So just the degree to which a color coded Excel spreadsheet with sensitive information is a little hard to follow, that sort of thing." 24-M |
| **Training and Technical Assistance: More Training** |  | "I've had to, every now and then, kind of re-educate the providers on what it is that I do as a care manager for Collaborative Care. Sometimes I get referrals that aren't necessarily anything to do with Collaborative Care, or I get people that are referred to Collaborative Care that are not candidates for the program. So sometimes just reeducating. I think that the providers look at me as a social worker when I'm here, so they kind of just want to reach for me for whatever problems they have to help out. And that keeps me from...if I have a full schedule of phone calls I need to make, or referrals I need to reach out, that keeps me from doing what I have to do because now I'm involved in something that has nothing to do with Collaborative Care." 10-A |
|  |  |  |
|  |  | "I would say just more education for the physicians on why [Collaborative] care is important. Then also, it would be nice to see...like tips or best practices or best referral tips for us to be able to share with our practices...I think if OMH released more stuff [resources] geared towards physicians...I think that would be nice." 31-I |
|  |  |  |
|  |  | "I think even if there was someone that just reached out to the different folks [clinics] that have CoCM and checked in with that care manager to say like, "Hey, are your needs met, do you need any additional support?" Kind of like Collaborative Care for the care manager. I think that would be something that would be nice." 07-I |
|  |  |  |
|  |  | "If there could be more training in education programs for mental health providers in primary care schools about how there are these multiple levels of types of care, I think it would probably help with buy-in and comfort over time. It's not that there's just like therapy and then there's medical, there's medical therapy, there's Collaborative Care, there's this other model of care. And so the more that I think behavioral health providers and primary care providers are trained in what this is, I think the easier it will be to create that mental shift for us to fully understand and buy into the program." 08-M |

Appendix 2: Comparison of New York State Medicaid Psychotherapy, Medicaid Collaborative Care, and Medicare Collaborative Care Billing by Primary Care Clinic Type

|  |  | **New York State Primary Care Clinics** | | |
| --- | --- | --- | --- | --- |
|  |  | **Private Practice** | **Hospital-Affiliated Clinics** | **FQHCs** |
| **Billable Service** | |  |  |  |
| **Medicaid Psychotherapy** (behavioral health service; includes direct services only) | **BH Providers** | - Licensed master’s level BH clinician and above | - Doctoral level BH clinician - Licensed Clinical Social Worker (LCSW) with certain patient populations | - Licensed Clinical Social Worker and above |
|  | **Patients** | - All patients | - Doctoral level BH clinician: all patients - LCSW: children, adolescents, and pregnant patients | - All patients |
|  | **Rates (21)**4/15/2024 1:06:00 PM | - 30 minutes $57.93/session - 45 minutes $76.45/session - 60 minutes $111.90/session | 30 minutes $54.26/session  45 minutes $70.39/session  60 minutes $105.07/session | - 30 minutes $57.93/session - 45 minutes $76.45/session - 60 minutes $111.90/session |
| **Medicaid Collaborative Care**  (primary care service; includes direct and indirect services) | **BH Providers** | - Master’s-level BH clinician **licensure** c**andidate / trainee** and above, (e.g. LMSW) - Registered Nurse (BSN recommended) - Nurse Practitioner | - Master’s-level BH clinician **licensure** c**andidate / trainee** and above, (e.g. LMSW) - Registered Nurse (BSN recommended) - Nurse Practitioner | - Master’s-level BH clinician **licensure** c**andidate / trainee** and above, (e.g. LMSW) - Registered Nurse (BSN recommended) - Nurse Practitioner |
|  | **Patients** | - 12 years and up | - 12 years and up | - 12 years and up |
|  | **Rates (21)** | Year 1   - Case rate $112.50/month   Year 2   - Case rate $101.00/month | Year 1   - Case rate $112.50/month - Retainage $37.88 /month (available after 3 months of patient enrollment)   Year 2   - Case rate $101.00/month - Retainage $25.25/month (available after 3 months of patient enrollment) | Year 1   - Case rate $112.50/month - Retainage $37.88 /month (available after 3 months of patient enrollment)   Year 2   - Case rate $101.00/month - Retainage $25.25/month (available after 3 months of patient enrollment) |
| **Medicare Collaborative Care**  (primary care service; includes direct and indirect services) | **BH Providers** | - A designated individual with formal education or specialized training in behavioral health (including social work, nursing, or psychology), working under the oversight and direction of the billing practitioner (primary care provider) | - A designated individual with formal education or specialized training in behavioral health (including social work, nursing, or psychology), working under the oversight and direction of the billing practitioner (primary care provider) | - A designated individual with formal education or specialized training in behavioral health (including social work, nursing, or psychology), working under the oversight and direction of the billing practitioner (primary care provider) |
|  | **Patients** | - All patients | - All patients | - All patients |
|  | **Rates (21)** | - 70 minutes initial month   - $147.12/month - 60 minutes subsequent months   - $139.18 - 30 min additional CoCM for initial or subsequent months (maximum of two uses per month with 70- and 60-minute codes only)   - $56.53 | - 70 minutes initial month   - $90.58/month - 60 minutes subsequent months   - $98.85/month - 30 min additional CoCM for initial or subsequent months (maximum of two uses per month with 70- and 60-minute codes only)   - $39.67 | - 70 minutes for initial month and 60 minutes for subsequent months   - $143.15/month |

Appendix 3: Interview Guide

***ALL INTERVIEWS***

1. Can you give me the name of your clinic and a general overview of what your role is in your clinic?
2. Can you describe the type of services offered at your clinic?
   1. PROBE- What does a typical primary care visit look like at your clinic?
   2. PROBE- any special populations, like pediatrics, OB-GYN, substance abuse etc.
   3. Ask specifically if they offer behavioral/mental health services (if not included in their initial response)
3. ***If they offer behavioral health services****:* You mentioned that you offer behavioral/mental health services in your clinic, can you give me an overview of what behavioral health care looks like at you clinic?
   1. PROBE- Do they screen? With what screeners? When does screening occur? By who?
   2. PROBE- do they refer out to a community mental health clinic?
   3. PROBE- Are Behavioral/Mental health providers located in a different area of the clinic, such as a different floor?
4. Thinking about your clinic population and the services you offer, could you describe any challenges that come with providing care in a clinic like yours?
5. Thank you for your participation in this interview, your responses are greatly appreciated. Do you have any questions for me or any last things that you would like to say? [WAIT FOR RESPONSE] Great, thank you so much, have a good day [END INTERVIEW]

***NEVER REACHED***

*Defined as never being in contact with OMH about CoCM.*

1. OMH has been offering the CCMP in some form since 2014, and our records indicate that your clinic has never had discussions with OMH regarding Collaborative Care or the CCMP program. Could you comment on your clinic’s knowledge of the CCMP/ COCM?
   1. ***If they never had knowledge*** *of it ask if t*hey have knowledge of other models of behavioral health integration (which is just offering behavioral/mental health services in primary care), such as Primary Care Behavioral Health (PCBH).
   2. ***If they never had knowledge*** *of it, ask* if they think COCM/CCMP may or may not be a good fit in their clinic and why they think this.
   3. **I*f they had knowledge,*** *ask about* reasons why engaging with OMH about Collaborative Care or the CCMP was not a priority/ was never pursued for your clinic?
      1. PROBE- competing priorities
      2. PROBE- did they even have capacity to offer behavioral/mental health services
      3. PROBE- patient population (low burden population OR population affected more by another ailment ex: diabetes etc)
2. Is there anything that could have been done differently to get your clinic to engage with OMH regarding the possible implementation of CoCM/CCMP?

***REACHED, NEVER ADOPTED***

*Defined as ever having contact with OMH with interest in CoCM. Either they reached out to OMH, OMH reached out to them or their organization as a whole.*

1. OMH has been offering the CCMP in some form since 2014, could you comment on how you got connected with OMH and the CCMP?
   1. What information did you gather from that connection? Was it about COCM or Just CCMP?
2. Can you elaborate on why your clinic was interested in CoCM and/or CCMP program
   1. PROBE- external or internal factors, - policy changes, new regulations, decision making processes, staff or patient demand, clinic goals, etc.
   2. PROBE- was the interest in CoCM/CCMP there before or only after connection with OMH?
   3. PROBE- was their organizational support for implementing an integrated behavioral health model, such as COCM.
3. Can you comment on any challenges your clinic may have had in getting connected with OMH about COCM/CCMP
   1. PROBE- lack of staff engagement, time pressure/competing priorities, OMH difficult to get ahold of, etc.
4. Can you comment on the impact that getting connected with OMH had on your clinic’s decision to further pursue CoCM/CCMP or not?

In order to help clinics get everything in place in order to bill for the CCMP (and thus implement CoCM) Training and Technical Assistance, such as coaching and training that focused on how to use a registry or finding a psychiatric consultant (among many other topics) was offered to clinics. Can you comment on your clinic’s knowledge of this TTA?

***If they knew about TTA***- Can you elaborate on why your clinic chose not to pursue TTA?

***If they knew about TTA***- ask who and how the decision to not receive TTA was made, group choice? One person? Organizational decision?

***If they knew about TTA***- ask whether they still plan to implement CoCM/CCMP

***If they did NOT know about TTA***- If you had known TTA was offered would your clinic have pursued receiving TTA? Do you think having TTA would have helped your clinic implement CoCM and bill CCMP?

After your clinic was in contact with OMH regarding possible CoCM/CCMP implementation, can you comment on any changes that may have occurred at your clinic?

PROBE- changes in clinic leadership, high turnover rates, restructuring of clinic, budget issues, etc.

Can you comment on how financial considerations may have played a role in your clinic’s decision to not pursue TTA for CoCM/CCMP?

There are many different ways and models of integrating behavioral health services into primary care, can you comment on whether your clinic ended up using or adopting a different behavioral health integration program?

If yes, which one and why?

PROBE- what specifically about CoCM/CCMP did not align with your clinic, what was the issue your clinic had with COCM/CCMP?

PROBE- what about [their clinic’s integration model] made it the best choice for your clinic to implement?

PROBE- who made the decision in your clinic on what model to implement

What would you say is the primary reason your clinic didn’t obtain TTA for adopting CoCM/CCMP?

PROBE- What were the major challenges to getting TTA?

PROBE- Internal factors (ex. organizational support, time, etc.)

PROBE - External factors (ex. changes in regulations or policy landscape, etc. )

- 1. Thinking back to when your clinic was discussing about whether or not to receive TTA for your clinic, can you think of anything that OMH could have done differently that might have made your clinic more likely to receive TTA? PROBE- get at factors other than internal here, so was OMH flexible with training scheduling etc.

***ADOPTED, NEVER IMPLEMENTED***

*Defined as ever having contact with OMH with interest in CoCM. Either they reached out to OMH, OMH reached out to them or their organization as a whole.*

In order to help clinics get everything in place in order to bill for the CCMP (and thus implement CoCM) Training and Technical Assistance, such as coaching and training that focused on how to use a registry or finding a psychiatric consultant (among many other topics) was offered to clinics. Can you elaborate on any Training and Technical assistance you received in relation to implementing CoCM/ the CCMP?

PROBE- was the training from OMH? Outside OMH?

PROBE- to your best memory, what were some of the topics discussed, and what topics/trainings were most useful to your clinic? Least useful? Is the information you obtained in those sessions still useful to you today?

PROBE- ask about if they felt that had the right amount of TTA, too much? Too little? Just right?

PROBE- cost of training to clinic? (if any), including time away from clinical duties.

Can you comment on the decision to pursue TTA for your clinic?

PROBE- Who in your clinic was responsible for deciding to pursue TTA, group decision? Was there organizational support? Provider support?

Can you discuss what TTA looked like at your clinic?

PROBE- Who received it?

PROBE- Are they still receiving support? If so what does that look like now?

Could you discuss some of the benefits or challenges of getting training and technical support?

PROBE- what about the TTA/ asking for TTA was easy and useful

PROBE- what was challenging/a barrier/ could have been better

Can you talk about the process of getting everything in order to submit a CCMP billing application?

PROBE- how long did it take? Was it easy/hard?

PROBE- what was the most challenging aspect of the billing application to get in place? Why do you think it was?

PROBE- Can you comment on the support/communication your clinic received from OMH in regards to completing the CCMP billing application?

Can you comment on challenges your clinic faced that inhibited your clinic from reporting outcomes to OMH?

PROBE- was your clinic still trying to provide COCM? Low patient interest? Lack of organizational support? Provider support?

PROBE- were there financial challenges? Workforce shortages ( they struggled to find a consulting psychiatrist/behavioral health care manager/etc)

Thinking back to getting the component of CoCM in place in order to apply to bill for CoCM, can you comment on what supports you received that were helpful to your clinic?

Supports from OMH, organizational, other

PROBE- What supports do you think your clinic needed that would have allowed your clinic to submit data to OMH?

***IMPLEMENTED, NEVER MAINTAINED***

*Defined as clinics that were in contact with OMH, received TTA (training and technical assistance), and submitted at least one quarter of data*

1. Can you talk about the process of getting everything in order to submit a CCMP (Collaborative Care Medicaid Program) billing application?
   1. PROBE- How long did it take? Was it easy/hard?
   2. PROBE- What was the most challenging aspect of the billing application to get in place? Why do you think it was?
   3. PROBE- Can you comment on the support/communication your clinic received from OMH in regards to completing the CCMP billing application?
2. ***If they submitted quarterly data****:* Thinking back to when your billing application was approved, can you discuss your clinic’s process of submitting monthly/quarterly data to OMH?
   1. PROBE- Time commitment/was it time-consuming? Easy/challenging to complete each quarter/month?
   2. PROBE- Can you comment on whether your clinic used/reviewed the metric data you had to submit to OMH for your own internal clinic purposes? Were these metrics helpful to you, did they drive change in your clinic? What metrics were most useful to your clinic?
   3. PROBE- Are there any other metrics you use in your clinic that you find helpful that are not reported to OMH?
3. ***If they submitted quarterly data****:* Thinking about submitting data to OMH, can you comment on what helped your clinic begin to report data to OMH, were there key facilitators that helped your clinic succeed in providing data?
4. PROBE- Internal clinic factors, external factors, TTA, IT systems, etc.
5. PROBE- Barriers both internal and external (ex. IT systems, lack of physician engagement, competing priorities, reimbursement issues, etc.)
6. ***If they submitted an application but never submitted data:*** Can you comment on challenges your clinic faced that inhibited your clinic from reporting outcomes to OMH?
7. PROBE- Was your clinic still trying to provide CoCM? Low patient interest? Lack of organizational support? Provider support?
8. PROBE- Were there financial challenges? Workforce shortages (ex. they struggled to find a consulting psychiatrist/behavioral health care manager/etc.)
9. PROBE- What supports do you think your clinic needed that would have allowed your clinic to submit data to OMH?
10. Thinking about actually billing Medicaid, can you comment on how easy or hard that process is?
    1. Supports from OMH, organizational, other that helped you bill?
11. Can you elaborate on the challenges your clinic faced in providing data to OMH/providing CoCM in your clinic?
    1. PROBE- What support do you think your clinic could have benefitted from to help your clinic sustain CoCM that was not available?
    2. PROBE- Can you comment on what your clinic offers now in terms of behavioral health services? Do you think you will be able to offer CoCM in the future? Why/why not?

***MAINTAINED***

*Defined as clinics who maintained CoCM/CCMP for at least 1 year post implementation*

1. It looks like your clinic has been able to submit data to OMH for at least one year, can you comment on why you think your clinic has been successful in continuing to submit data for a year?
   1. PROBE- what supports did they have that helped? Challenges they had to overcome?
   2. PROBE- are they still providing CoCM/submitting data to OMH?
      1. ***If not***: Why not? What specific challenges came up that forced you to stop submitting data/stop providing COCM?
      2. ***If not***: What support do you think your clinic could have benefitted from to help your clinic sustain CoCM that was not available?
      3. ***If not***: Ask them to comment on what their clinic offers in terms of behavioral health services. Do they think they will be able to offer CoCM in the future? Why/why not?
      4. I***f yes:*** ask them to comment on what challenges they have faced in continuing to provide CoCM/provide data to OMH? What support have they received that helped them continue to provide COCM?
      5. ***If yes:*** Do you think your clinic will be able to continue to provide CoCM in the foreseeable future? Why/why not, what supports do they have/think they will need in order to continue to provide COCM?

Appendix 4: Qualitative participant demographics.

| **Clinic Code** | **RE-AIM step** | **Clinic Type** | **Rural** | **Participant's Role in clinic** | **Services offered** | **Screeners used** | **Who administers screenings** | | **Frequency of screening** | **External referral process** |
| --- | --- | --- | --- | --- | --- | --- | --- | --- | --- | --- |
| **NYS-01** | Maintained | Private Practice |  | Chief Operating Officer | family practice (birth to death), any type of primary care visit (physical, gynecological, office visit, etc), 30% pediatrics, 30% geriatric | GAD-7 | social worker |  | | But once it goes beyond where they need extra help, or counseling's not working along with the medications that are prescribed, we refer out a lot. it's up to provider discretion, but usually it's if they've had the trial treatment of a primary med. Usually three to six months and if they don't see any improvement for ADHD, it's definitely six months or more on a med for depression. And if that's still not improving, then the referral goes out. So, it's probably, I'd say, if it's not working within six months, then they definitely get the referral out.NYS_21 U |
| **NYS-03** | Adopted, Never Implemented | Private Practice |  | Division Lead for Psychiatry | substance use, primary care, **child and adolescent psychiatry (3+), adult psychiatry, therapy,** neurology, endocrinology, ophthalmology, cardiology, radiology, lab, urgent care, family medicine, pediatrics, geriatrics | electronic PHQ-9, GAD-7, CAGE, AUDIT | patient on phone, PCPs | "Everybody gets an annual PHQ-9...We do the CAGE and the audit for substance use disorders. That's done annually by the PCPs." | | So for the most part everything's in house, besides for those emergent situations, we're just not set up to kind of care for that, so we do provide like the crisis hotline and other areas that they can go if something was to arise when we're not necessarily available. Outside of that, we do refer out to a variety of those same things or other behavioral health clinics if it's somebody who does not qualify within our patient criteria,-NYS_41 U |
| **NYS-04** | Adopted, Never Implemented | FQHC |  | Chief Health Integration Officer | full ambulatory care health center, adult, OBGYN, pediatrics, cardiology, urology, surgery, podiatry, ophthalmology, nutrition, **BH component,** HIV wellness program | PHQ-2, PHQ-9, GAD-7, CRAFT, AUDIT-C, DUST, EXPERT, SDOH, Colombia Severity Rating Suicide Scale, HIV | medical assistant | "[PHQs are] done at every visit as compared to every 12 months. In fact, all those screenings are done at every visit except the SDOH. So the PHQ-2, PHQ-9, your GAD-7, and for the younger population, we use the craft. And then we use the AUDIT-C and the dust and expert for alcohol and drug counseling and referrals. And based on if a patient is expressing any potential suicidal ideations or any suicidal risk, we use the Columbia Severity Rating suicide scale. It's built into the progress note that you just drop it in and you can complete it, which can then alert the behavioral health team that they need to do some active involvement....The social determinants of health. That's when it's supposed to be done once a year " | | So a lot of the times we refer out for [substance use services]-NYS_32 U |
| **NYS-05** | Adopted, Never Implemented | FQHC |  | Chief Medical Officer | mostly primary care; specialists: **BH specialists,** psychiatric prescribing clinicians, optometry services, podiatry, OBGYN, dental, nutritionist; rest of staff is family medicine, internal medicine, and pediatric providers; some **medication assisted treatment, licensed prescribers** | PHQ-2 triggers a PHQ-9, substance abuse screening, Edinburg for postpartum depression, MCHA, ACEs screening, adolescent wellbeing screener, depression screener, suicide, drug and alcohol screening, PHQ-9 | medical assistant, BH specialist |  | | We only refer...Pre-pandemic, I would say once every 10 patients, but now during the pandemic, probably three or four out of 10 patients need mental health referral. Yeah.-NYS_27 U |
| **NYS-06** | Maintained | Private Practice |  | Pediatrician | pediatrics (birth to 26 y/o) | PSCS (anxiety, depression, conduct disorder, oppositional defiant), depression screening, anxiety screening, alcohol screening, Vanderbilt, SCARED, PHQ-9 | "We now use a program called CHADIS. Where they cover a lot of screening forms, and complete forms before they come in.", electronically, used to be front desk | "We start screening very early from the baby PSC to a toddler PSC. And once you're 12, we start screening specifically for depression, anxiety, alcohol use as well as the PSC. So there four screenings we do once you're a toddler, and up....before the pandemic I used to screen all teenagers but they don't come often. So when they come I just screen them. But the younger kids is when they have their checkups but for teenagers we screen them at any visit we see them just because they don't come to doctor's office. But with the pandemic, and our transitions... Before we used to use a lot of paper screening more reliable but with the pandemic we've switched the electronic screening just reduce the amount of time they in the office with exposure." | | we have to refer out...So we have to refer out, because our practitioners here, our healthcare practitioners, mental health isn't their primary. it's really on an as-needed basis-NYS_13 U |
| **NYS-07** | Implemented, Never Maintained | Private Practice |  | Behavioral Health Care Manager | primary care, family practice (infants to geriatric), **BH services** | PHQ-9 and GAD-7 "[if] the screen has coming up positive, then I kind of step in and introduce the program (CoCM) to them", bipolar disorder screener (CIDI or MDQ), OCD screener (Yale-Brown OCD scale), GAD-2, Whoolley | BHCM, doctor | "Doctor will, especially if it's a newer patient, she'll tend to do the anxiety depression screening questions with them initially. And then if she identifies that their score is over a 10 or higher, or even if their scores are low and she still has an inkling of concern for their symptoms, she still calls me in and we kind of go over...the program"i  bipolar, anxiety, depression mostly/frequently, rarely OCD, GAD-2 and Wholley for every patient (if positive then PHQ-9 and/or GAD) | | We have a lot of patients with longterm needs that are really referred out and on wait lists to get in-NYS_59 R |
| **NYS-08** | Maintained | FQHC |  | Chief Behavioral Health Officer | **integrated** medical, dental, and **BH care;** dental, primary care services, social work care management, nurse care management, refugee health program | PHQ-2, PHQ-9, GAD-7, Colombia Suicide Severity Rating Scale, DAST-10, AUDIT C, UDS depression remission measure | nursing staff, either LPN, medical assistant, or RN, depending on who's available | "We do have the GAD-7 for whenever a patient has an anxiety disorder diagnosis and the primary care provider wants to check on their symptoms, or if there's some additional stressors noticed, or the patient seems a little on edge or nervous, they might just do it as if that's as needed. There's not a regular practice of screening with the GAD, we also screen with... We have the Columbia Suicide Severity Rating Scale, but it's not really utilized very heavily. We have a pretty good clinical interview process that we do around safety assessment. And then we have single question screeners for intimate partner violence and human trafficking. And then substance use, we have the craft, we have the DAST-10, the AUDIT C." | | Less than 5% of cases are referred out.NYS_14 A |
| **NYS-10** | Adopted, Never Implemented | Hospital- Affiliated Clinic |  | Behavioral Health Care Manager | **CoCM,** health home services, case management services, primary care services, adult only (18+), gynecology, | GAD-7, PHQ-9, CAGE | nurses | "At least the annually. I mean, if a patient is coming in for something strictly like a sick visit or something that's not necessarily a physical or a Medicare wellness, then they don't get screened, but for the majority... I think our last, we went from... When I first started collaborative care, I think they were screening 16% of patients and now it's 90 something." | | We try to keep them in-house as much as we can. If we feel like we have the capacity to help them. But anyone who has a mixed picture like substance abuse with behavioral health, they get referred out to a special program, a different program....Our providers are a little shy when it comes to medications and treatment that way they. They tend to want to hand them off to the psychiatrist but it becomes a bottleneck because we don't have as many psychiatrists as we have all the other-NYS_05 A |
| **NYS-11** | Maintained | FQHC |  | Behavioral Health Care Manager Coordinator/Supervisor | primary care, OBGYN, dental, **BH care management,** care coordination, chronic disease management (can't provide all these services in all their clinics because staffing is an issue) | PHQ, GAD | BHCM? | "would we either in annual assessment, patient complaints, follow up, whatever, those kinds of things, so that's streamed through primary care, and then linked through warm handoffs and referral processes to interim behavioral health care management, and then you do what we need to do." | | We refer it to our local hospital,they have an in-depth psychiatric program - NYS_04 A |
| **NYS-13** | Never Reached | FQHC | Rural | Ambulatory Care Manager | family-based health center, **"low-grade" behavioral issues / MH challenges**, everything from 6mo of age to elderly | depression screening (PHQ-9) | RNs | "We do have a screening that we do for every patient, whether they're a diagnosis of mental health or if they're just a new onset so that we're not letting anybody slip through. We have a depression screening that we do for every patient, and then if it's triggered, we go more in-depth....for a typical patient, it's every three months. Some of the patients that we only see once a year or twice a year, that happens at their visits as well. If they are high alert, it's every visit." | | Right now we're really short staffed. We're doing a lot of referring out to other sites or other clinics-NYS_43 A |
| **NYS-14** | Adopted, Never Implemented | FQHC | Rural | Chief Operating Officer of Behavioral Health | primary care, internal medicine, pediatrics, BH, dentistry, urgent care dept., OBGYN, cardiology, endocrinology, infectious disease, care management dept., WIC provider for OC, HIV/AIDS, homeless health organization, **behavioral and mental health services** | depression screening, alcohol and addiction screening, SBIRT, "two question depression screening is given in the registration package, and if it's responded to positively then it's picked up from there by the MA who will then do the full screening" (PHQ-2 and PHQ-9) | medical assistant | "Established patients have to have a depression screening at least once a year." (PHQ-9) | | we do have a lot of patients that have more acute mental health that need to be referred out. -NYS_10 A |
| **NYS-16** | Adopted, Never Implemented | FQHC |  | Director of Behavioral Health | **integrated** (sort of) **BH care,** urgent care | PHQ-9 |  |  | | I would say 30 to 40% of those we probably refer out, like as just an immediate we know we can't get them in.-NYS_03 A |
| **NYS-17** | Implemented, never Maintained | Hospital- Affiliated Clinic |  | Manager of Outpatient Social Work | **CoCM**, primary care, OBGYN, pediatrics, outpatient oncology, medical social work services, **some specialized BH services** | PHQ-9, GAD-7, Edinburgh, MDQs, Columbia’s, SCARED | LPN or MA | "At a minimum annually for PHQ-9. Then from there, it's really, again, just provider comfort level as indicated. It's really the social workers' role to be screening them once they're enrolled in collaborative care. We are doing it monthly with anyone who's enrolled in collaborative care. But outside of that, in terms of generalities, it's probably at a minimum annually and then, as it comes up. Some providers use it as a tool when they're assessing, and some providers use it as a, we have to do this once a year. Then you just throughout the year, tell me how your symptoms are, as opposed to formally tracking it." | | We do refer to outpatient mental health.when the behavioral health specialist evaluates the patient decides that the patient needs a higher level of care than what they can offer. But sometimes it's a long wait list or sometimes the patients just don't feel like it's a good fit with the therapists that they have been assigned to. -NYS_31 I |
| **NYS-21** | Never Reached | Private Practice | Rural | Practice Manager | kids 0-21 y/o (just pediatric practice), primary care mostly, **behavioral health screenings** | PHQ-9, CRAFFT, SCARED, Vanderbilts, One-Question Screen in AUDIT, GAD | "The parents will fill them out. Or in Vanderbilts cases, the teachers and then the providers will review it either before the appointment or in the appointment or after, depending on what transpires at the appointment. But it's directly with the providers." | "So, 12 and up, we do the PHQ-9 and CRAFFT screenings on everyone, even if they're not doing any complaints of anxiety or depression. If they are having anxiety, we will give them the SCARED forms and evaluate that way."  "At least annually, with the PHQ-9 and the CRAFFT and the One-Question Screening for 18 and older. Otherwise, the anxiety and more depression screenings are just as needed. So, when you get the call. "Oh, I'm concerned with X, Y, Z," and then we'll give it to them at the front desk when they come in." | | There are some cases where the patient is, maybe we've tried them on multiple medications and the treatment is just not really getting them to where they're improving or doing much better. Some patients ... like for a doctor here in the office, she's very clear about really not wanting to go the route of the more addictive medications like Xanax or any heavy medications. So usually if patients are continually asking for Xanax or we're just noticing that the treatment, the things that we've tried with our psych consult and things like that are just really not helping and not effective, that's when we try to refer out to a more local organization that has the capacity to do counseling and med management.- NYS_07 I |
| **NYS-23** | Implemented, Never Maintained | FQHC |  | Behavioral Health Care Manager Coordinator/Supervisor | OBGYN | PHQ-9, GAD | providers |  | | There is some referring out-NYS_30 I |
| **NYS-24** | Maintained | Hospital- Affiliated Clinic |  | Medical Director of Depression Care | **mental health services,** primary care (adult patients 18+), substance abuse services; only have 20-30 mins for visits--"we have 20 to 30 minutes for the visits and it can be hard to address all those things during the visit" | PHQ-9, GAD-7, alcohol and substance abuse, PHQ-2 (if positive then PHQ-9) | depression care managers, medical assistant | "it is actually our two depression care managers who decide sort of which arm of the program the patient should go into, whether it seems like they're appropriate for therapy and meet the requirements of PHQ-9 store and GAD score and stuff like that, or whether the patient is more interest in medication. So they should go to the evaluation clinic, or whether they're more complicated and need the psychiatrist."  "For depression, every patient gets a PHQ-2 annually, so it's sort of on a rotating 365 day thing. And then if the patient is positive for the PHQ-2, they're given a PHQ-9. The patient would also be screened if they're sort of exhibiting symptoms of depression or anxiety, even if it sort of doesn't fall at that one year mark. And then for substance use, it's sort of inconsistently done for all patients at sort of inconsistent times, maybe a year. And if it's during one of the therapy visits, the therapists are doing it at every session. And so are we in the evaluation clinic. So for the sort of behavioral health components, it's those the PHQ-9 and the GAD-7 are being done at all visits, but for the PCP visits it's just annually." | | I would say 99.9% of the time it's in-house. Then if somebody needs to be referred out, its usually because it's for emergency services being referred to because of a crisis suicidal ideation, homicidal or something, something a psychotic disorder that is brewing, but most of time we're able to handle the cases in-house.-NYS_29 I |
| **NYS-26** | Maintained | Hospital- Affiliated Clinic |  | Administrator | care for all ages, maternity care, preventative care, some social work support, **psychiatrist but not CoCM anymore,** primary care, hospital is specialist focused |  |  |  | | if somebody is deemed that they have severe mental health issues or would need more intensive services, then they're referred out.-NYS_52 I |
| **NYS-27** | Never Reached | Private Practice |  | Medical Assistant | pediatrics primary care | PHQ-9 |  | "every teenager that comes in does a PHQ-9...Whenever they come in for their physicals. Any 13 and older get the form." | | If they have needs outside of what collaborative care can offer, we will link them with appropriate resources, either within our system or elsewhere, depending on what the patient preferences and what the need level is.We try to keep everything in-house. But sometimes you have patients who either live further away or they prefer certain places. Really, our goal is to link them with whatever's most appropriate.-NYS_17 I |
| **NYS-29** | Implemented, Never Maintained | FQHC | Rural | Senior Director of Behavioral Health | primary care, pediatrics, women’s health, prenatal, OBGYN, dental, podiatry, optometry, nutrition, WIC, psychiatry, HIV, PrEP services, advocacy services, substance use (medication assisted treatment), **mental health services**, all ages | depression and anxiety screeners, substance use disorders, PHQ-9, GAD-7, AUDIT, DAST, CRAFT, other screening tools depending on age | LMSW | "So we use our PHQ-9 and GAD-7 is treat to target tools and do we get the baseline, usually done as part of that medical visit that either is a push in by the LMSWs or pull in through that warm handoff process. So then we readminister the PHQ periodically or GAD periodically from there." | | there's a fair amount of referring out mostly for psychiatrists just because ours is sort of... There's one of her. We have 20,000 patients and she's only here two days a week. So for any patient who needs a long term psychiatrist. And similarly for any patient who wants the long term therapy, because our therapists are trying to see patients sort of on the four to six month period range, and then they would be referred out as so.-NYS_24 M |
| **NYS-30** | Implemented, Never Maintained | FQHC |  | Director of Process Improvement and Analytics for Behavioral Health department | **BH services with psychiatric care, CoCM,** primary care, family health, HIV/AIDS, sex/human trafficked services, undocumented services, diabetes, case management, care navigation services, substance abuse | PHQ-9, GAD-7, DAST, AUDIT, ADHD, DLA, psychosocial assessment, bipolar screener, PTSD |  | "We are big fans of a PHQ-9 and of the GAD-7. Those are initial screeners. Everybody will get one of those or both depending on symptom presentation and then follow up accordingly....[the screening] happens an initial first visit for sure. It'll be a lot of, we usually will frame in behavioral health land anyways, we're going to ask you a lot of questions. Here we go. Buckle up. Then based on somebody's diagnosis, we strive to do like a PHQ-9 for depression diagnosis at least once a month, if not at every visit. Then of course, if there's any suicidal ideation or risk, there's due to the safety plan, the CSSRS risk assessment, those happen every session if you're somebody who has active suicidal ideation. But they have been depending on your diagnosis, right. We do PHQ-9s for somebody with bipolar or somebody who's eight, but they happen, I guess, contextually as needed, but definitely at least once." | | often our patients with severe and persistent mental illness, things like psychotic disorders, bipolar disorder the ones that really need medication management primarily, or ADHD, any controlled substances those we tend to have to refer out for psychiatry. -NYS_08 M |
| **NYS-31** | Implemented, Never Maintained | Hospital- Affiliated Clinic |  | Administrator | hospital system with several clinics; they specifically are a family care office (see from birth to senior), primary care, **CoCM,** case management, Suboxone provider within network | PHQ, GAD, substance abuse questionnaire, M-CHAT, AUDITS | BH specialists | depression and anxiety: "The primary care offices themselves are screening for depression and anxiety once a year on all of our patients that would fall in the denominator. But I think the behavioral health specialists in particular are using those two centers. Once a primary care patient is referred to the behavioral health specialist, then they're using them on a more regular basis, I think, than the primary care office. For the most part, the primary care office is doing once a year, or as they see fit. If a patient comes in and says, "Oh, I'm having depression feelings or whatever, anxiety feelings." I think they would use the screener at that point.."  for substance abuse: "At least once a year it's a physical." | | So we can do some further assessment and determine what it is that the patient needs and what the best place is for them to go. We try and keep as much in-house as possible just because we know we have better access. And we also have much better collaboration.-NYS_08 M |
| **NYS-32** | Never Reached | Private Practice | Rural | Certified pediatric primary care nurse practitioner | pediatrics primary care, some newborns, preventative care, acute care, chronic care management, **mental health counseling**, | "we do a lot of screening...there is some screening tools related to substance abuse that sometimes we will use, but very infrequently", GAD-7, Vanderbilts, Conners rating scales, CRAFT, PHQ-2, PHQ-9, ADHD, SCARED; "In the last few years invested in what's called CHADIS, I don't know if you've heard of this program, but it's another software program that interfaces with our electronic medical record. We have eClinicalWorks. And what this does is you can set up certain visit types to link with certain screening tools. So in the last few years, this has been huge, although of course an IT nightmare...And when you look at CHADIS, the list of questionnaires is daunting. It's pages upon pages upon pages" | providers |  | | our providers will see behavioral health issues and to the extent that they can manage it, they will, with medications. But then there might come that point in time when it is beyond them and we need to refer out to a psychologist, social worker, or a psychiatrist. - NYS_01 M |
| **NYS-38** | Never Reached | Private Practice |  | Executive VIce President | primary care mostly, **just started on psych piece,** subacute rehab, urgent care, small amount of adolescents and kids, skilled nursing, assisted living, pain management |  |  |  | | sometimes it is a patient's preference. Sometimes our clinics won't accept them and it is often it is for substances. So we prefer referring within our institution but for various reasons, location being one, some people prefer to get their substance use treatment at other facilities.-NYS_50 M |
| **NYS-41** | Never Reached | Private Practice |  | Office Manager | endocrinology, internal medicine, diabetes, thyroid, physical therapy, dietitian, good % are elderly (70+), **mental health services** | standard memory test, "I think we do [use depression and anxiety screeners], but I'm not exactly sure as to what they ask them. See, most of our elderly patients come with their children, their children come with them, so the children will know things that are going on and we'll talk to them after, about it. So, when there's screening, usually the child will know what to check. Whoever the caregiver is, the wife, the husband, the child, the niece, the nephew, whatever." |  | "I would say once a year, unless it's a very elderly, or if the child, or whoever the caretaker is, points out something different to us. So, we will do it usually after a complete physical. Some people have their complete physical, if they're younger, once a year, and for the others it can be for like twice a year, depending on their age." | | for anybody who has an acute mental health need, where we really want them to be set up with someone urgently...try and get them set up with mental health within the community.-NYS_47 M |
| **NYS-43** | Adopted, Never Implemented | FQHC | Rural | Director of Behavioral Health | primary care, dental, **BH,** family planning, care management, patient navigation, other support services | PHQ-2, PHQ-9, GAD-7 | medical providers | "So, when there's screening, usually the child will know what to check. Whoever the caregiver is, the wife, the husband, the child, the niece, the nephew, whatever." | | We also will link and refer to higher levels of care if necessary-NYS_11 M |
| **NYS-47** | Maintained | Hospital- Affiliated Clinic |  | Associate Medical Director | adult primary care | PHQ-2, PHQ-9, GAD-7 | provider | "For the PHQ-2, it happens at every single visit. Then for the GAD-7, it's up to the provider's discretion." | | So we have patients we feel that we can't manage, we have patients that screen for bipolar disorder, we have a patient that a paranoid, and how she's affected disorders.-NYS_06 M |
| **NYS-50** | Maintained | FQHC |  | Director of Integrated Behavioral Health | primary care, speciality care, **BH servivces, CoCM,** diabetes, underserved population services, substance use treatment center | depression, suicidality, and substance use screening, PHQ-2 then PHQ-9, GAD-7, AUDIT, DAST | medical assistant | "Right now it is at every visit. And we are working on what we call a smart screening project, which is to have an algorithm within our EMR that will indicate when a particular patient needs to be screened. of course it is annually for everybody, but more often for are people who have previously screened, positive people who are pregnant or have other risk factors." | |  |
| **NYS-52** | Implemented, Never Maintained | FQHC |  | Chief of Pediatric Community Health | pediatrics only clinic (0-21 y/o), **early childhood MH component;** community based partnerships: medical legal partnership, food stamp enrollment partnership, health insurance enrollment partnership, WIC location partnership | social needs screener, PSC-17, PHQ-2 | "they're given those screening tools when they check in and then it's on paper, they enter that with a pencil of either the parents or the child depend on the age of the child. If they're 12 and above, the child complete the mental health screeners themselves. If they're below that age, then with the parents." |  | |  |
| **NYS-59** | Reached, Never Adopted | Hospital- Affiliated Clinic |  | Manager | primary care, pediatrics, embedded case management, **embedded BHCMs**, labs + x-rays, substance abuse, hepatitis C, OBGYN, **CoCM** | PHQ-9, GAD-7 | BHCMs | "The regulation is to screen at least once a year for a PHQ-9 in adolescents 12 and up, into adulthood. And you do at least, at minimum, a PHQ-9 once a year. If the PHQ-9 is 10 or above, that may facilitate a referral to the behavioral healthcare manager. Many times the providers identify needs and refer to the behavioral healthcare manager without a PHQ-9 10 or above, or without a diagnosis of anxiety or depression. They just identify needs and send them along. We do, upon referral, look at that PHQ-9, GAD-7. I have requested that our behavioral healthcare managers do an expert screen on all new collaborative care patients and referrals, so they have implemented that this year and are doing that. Because they weren't doing it on everybody; they were doing it as like, "Oh, I've identified this might be a need." And I said, "No, no, we do it on all new patients."" | |  |
| **NYS-64** | Adopted, Never Implemented | FQHC | Rural | Senior Director of community relations, pharmacy affairs, and compliance office | primary care, pediatrics, family practice, substance abuse, diabetes, addiction |  |  |  | |  |
| **NYS-67** | Reached, Never Adopted | FQHC |  | Senior clinical social worker, Assistant Director of the mental health department | medication and therapy services (**MH services**), primary care | depression screening, PHQ, GAD-7AA, substance abuse, trauma care screening (ACE score) | care manager, PCP |  | |  |

Appendix 5: New York State Medicaid Collaborative Care claims data before and after CPT codes were added as a billing option


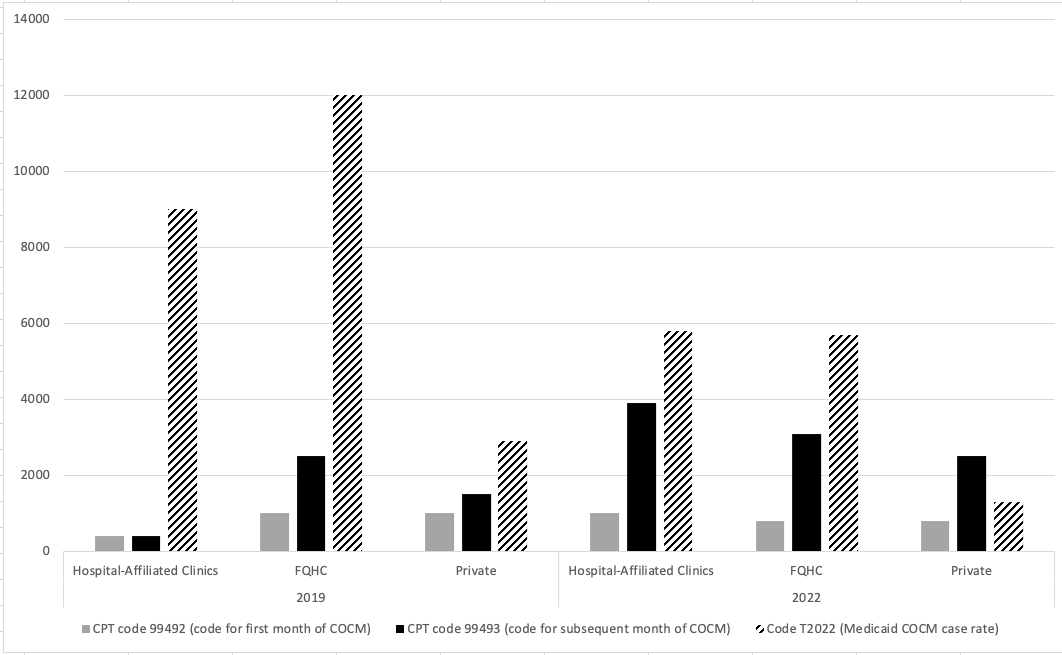


Appendix 6: Quantitative survey

New York State Behavioral Health Integration Survey

Welcome to the New York State Office of Mental Health's Behavioral Health Integration Survey!

This survey should only take you around 20 minutes to complete, and will go by even faster if you already have clinic data from 2019

Please remember to take this survey once PER CLINIC you are responding for.

As an incentive, upon completion of this survey you will be entered into a lottery to win a $500 gift card! Thank you!

Attached is a summary of the types of questions that are asked in this survey.

Reminder: IT WOULD BE VERY USEFUL TO HAVE YOUR CLINIC's ANNUAL REPORT AT HAND.

[Attachment: "Summary of Survey Questions .docx"]

What is the name of your clinic?

Is your clinic a Federally Qualified Health Center Yes

(FQHC)? No


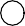

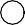

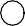

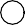


Is your clinic a Rural Health Clinic (RHC)? Yes No

Which one best describes your clinic? Part of a hospital system

Part of an Independent Physician Association (IPA) Private practice


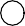

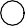

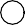


What is the name of the larger system to which your clinic belongs?

Please provide your best estimate on the breakdown of the following patient demographics at your clinic for fiscal year 2019, before COVID-19 (before March 1, 2020) PLEASE USE YOUR CLINIC'S ANNUAL REPORT :

How many unique patients visit your clinic annually? (not number of patient visits)

| What is your best estimate? 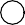 | 0-25% |
| --- | --- |
| 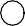 | 26-50% |
| 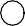 | 51-75% |
| 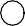 | 76-100% |
| What percent of your clinic population identifies as female? |  |
|  |  |
| What is your best estimate? 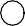 | 0-25% |
| 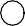 | 26-50% |
| 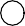 | 51-75% |
| 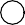 | 76-100% |
| What percent of your clinic patients are over the age of 18? |  |

| What is your best estimate? | 0-25% 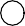 |
| --- | --- |
|  | 26-50% 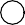 |
|  | 51-75% 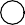 |
|  | 76-100% 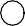 |
| What % of your clinic's patients are over the age of 65? |  |
|  |  |
| What is your best estimate? | 0-25% 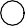 |
|  | 26-50% 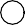 |
|  | 51-75% 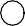 |
|  | 76-100% 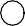 |

What % of your clinic patients identify as underserved or minority populations?

What is your best estimate? 0-25%

26-50%


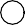

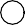

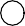

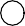


51-75%

76-100%

What % of your patients have a diagnosis of depression and/or anxiety

What is your best estimate? 0-25%

26-50%


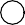

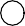

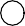

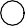


51-75%

76-100%


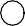

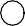


Does your clinic provide primary care services? Yes No

Please Describe what type of services you do provide at your clinic

The following is a list of potential prescribers in a clinic. Please check off all of the prescriber roles that your clinic currently has employed

please check off all of the prescriber roles that your clinic currently has employed (check all that apply):

Primary care provider (may include MD, DO, PA, or ARNP)

Psychiatric provider (MD, DO, PA, or ARNP specialty trained in psychiatry and does not provide primary care services)

Other Prescriber not captured above

Please describe the other prescriber(s) at your clinic that were not covered by the previous choices :

What is the total FTE of the primary care provider(s) (may include MD,DO, PA, or ARNP) at your clinic? (in

0.5 increments, e.g. 1.5, 2.0 etc.) (Example: 1.0 FTE equates to 40 hrs/week, 0.5 to 20 hrs/week. If your clinic has 2 providers who each work part time at 20 hours/week, then you would put down 1.0 FTE (0.5 plus 0.5))

What is the total FTE of the psychiatric provider(s)

(MD, DO, PA, or ARNP specialty trained in psychiatry

and does not provide primary care services) at your (Example: 1.0 FTE equates to 40 hrs/week, 0.5 to 20 clinic? (In 0.5 increments, e.g. 1.5, 2.0, etc.) hrs/week. If your clinic has 2 providers who each

work part time at 20 hours/week, then you would put down 1.0 FTE (0.5 plus 0.5))

What is the total FTE of the other prescriber(s) at

your clinic? (In 0.5 increments, e.g. 1.5, 2.0, etc.)

(Example: 1.0 FTE equates to 40 hrs/week, 0.5 to 20 hrs/week. If your clinic has 2 providers who each work part time at 20 hours/week, then you would put down 1.0 FTE (0.5 plus 0.5))

Does your clinic currently employ a Behavioral Health Yes Provider (BHP) (Counselor, social worker, marriage and No family therapist, psychology, or other master's level

non-prescriber)?


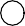

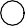

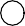

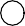


Does the behavioral health provider(s) (BHP) at your Integrated care team clinic work as a part of an integrated care team or Independently independently?

The next set of questions asks about your clinic's Insurance payer mix: Please provide answers to the following questions using data from fiscal year 2019, (before COVID-19)

What proportion of your patients have their primary payer as a commercial (private) insurance (e.g. Aetna, Blue Cross, etc.)

What is your best estimate? 0-25%

26-50%


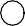

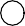

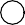

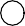


51-75%

76-100%

What proportion of your patients have their primary payer as Medicaire or Medicare Advantage ONLY (not medicaid)

What is your best estimate? 0-25%

26-50%


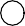

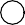

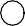

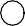


51-75%

76-100%

What proportion of your patients have their primary payer as Medicaid or a Medicaid Managed Care Organization ONLY?

What is your best estimate? 0-25%

26-50%


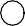

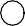

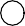

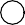


51-75%

76-100%

What proportion of your patients with Medicare are dually-eligible for Medicare and Medicaid?

What is your best estimate? 0-25%


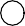

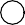

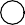

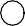


26-50%

51-75%

76-100%

What proportion of your patients have their primary payer as Indian Health service (IHS)?

| What is your best estimate? | 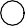 0-25% |
| --- | --- |
|  | 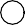 26-50% |
|  | 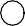 51-75% |
|  | 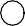 76-100% |
| What Proportion of your patients are uninsured? |  |
|  |  |
| What is your best estimate? | 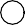 0-25% |
|  | 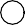 26-50% |
|  | 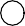 51-75% |
|  | 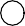 76-100% |

What proportion of your patients have a primary payer NOT mentioned above?

| What is your best estimate? | 0-25% 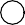 |  |
| --- | --- | --- |
|  | 26-50% 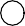 |  |
|  | 51-75% 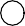 |  |
|  | 76-100% 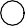 |  |
| Please describe this other payer: |  |  |
|  |  |  |
| Payer Mix Calculation |  |  |
|  |  |  |
| STOP! Edit your responses so that they add to 100%. |  |  |
| The following are a set of specific billing codes.  Please indicate if your clinic has ever used one or  more of these billing codes: 99492/G0512, 99493/G0512, 99494/G0511, or G2214? | Yes No 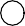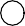 |  |
| Out of all of your CoCM and/or BHI services, which of | 1-10% 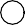 |  |
| the following best describes the proportion of | 10-20% 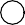 |  |
| services paid for via CoCM and/or BHI codes | 20-30% 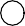 |  |
| 99492/G0512, 99493/G0512, 99494/G0511, and G2214? | 30-40% 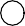 |  |
|  | 40-50% 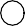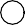 I don't know. |  |

The next set of questions is going to ask about how your clinic pays for Behavioral Health Services. If your clinic does not provide behavioral health services, you would simply answer "no" to the following questions. As a reminder- please provide answers to the following questions using data from fiscal year 2019, (before COVID-19)

Do you currently pay for BH services via Yes

fee-for-service psychotherapy codes? No


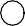

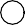


What proportion of your current BH services do you pay for via fee-for-service psychotherapy codes?

What is your best estimate? 0-25%


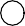

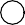

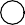

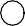


26-50%

51-75%

76-100%

Do you currently pay for BH services via Yes

fee-for-service direct psychiatry visit codes? No


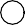

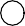


What proportion of your current BH services do you pay for via fee- for-service Psychiatry codes?

What is your best estimate? 0-25%

26-50%


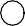

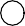

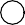

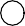


51-75%

76-100%

Do you currently pay for BH services via grant Yes

funding? No


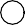

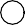


What proportion of your current BH services do you pay for via grant funding?

What is your best estimate? 0-25%

26-50%


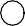

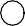

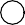

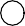


51-75%

76-100%

Do you currently pay for BH services via BH Yes

program-specific donations? No


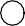

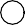


What proprtion of your current BH services do you pay for via BH program-specific donations?

What is your best estimate? 0-25%

26-50%


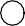

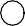

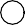

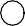


51-75%

76-100%

Do you currently provide BH services that are not Yes

billed or reimbursed for (i.e., subsidized by general No revenue)?


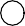

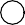


What proportion of your current BH services are not billed or not reimbursed (i.e., subsidized by general revenue)?

What is your best estimate? 0-25%

26-50%

51-75%

76-100%

Before you submit-the researchers on this project plan Yes

to conduct a few brief interviews with a portion of No

clinics responding to this survey. As an incentive,

those chosen for an interview will have their names entered 50 more times for the lottery to win a $500 gift card. Would you be interested in potentially being contacted for an interview?

*Please note that by clicking yes you will not be automatically chosen for an interview, and if chosen for an interview you can decline at any time

Please provide your name and email so we can send you a gift card.
